# Bioassay-guided fractionation and identification of wound healing active compounds from *Khaya senegalensis* leaves

**DOI:** 10.1371/journal.pone.0339051

**Published:** 2026-02-02

**Authors:** Akrimi Najihah Mohd Khamil, Norizamimie Nordin, Mohamad Afiq Aizuddin Rosdi, Mohamad Faiz Hamzah, Mohd Hasnan Mohd Noor, Siti Zuraidah Mohamad Zobir, Amyra Amat Sain, Mohammad Tasyriq Che Omar, Mohamad Nurul Azmi Mohamad Taib, Ayunni Salihah Zalaludin

**Affiliations:** 1 Malaysian Institute of Pharmaceuticals and Nutraceuticals, National Institutes of Biotechnology Malaysia, Gelugor, Penang, Malaysia; 2 School of Chemical Sciences, Universiti Sains Malaysia, Minden, Penang, Malaysia; 3 Institute for Medical Research, National Institutes of Health, Ministry of Health Malaysia, Shah Alam, Selangor, Malaysia; 4 Biological Section, School of Distance Education, Universiti Sains Malaysia, Minden, Penang, Malaysia; Kerman University of Medical Sciences, IRAN, ISLAMIC REPUBLIC OF

## Abstract

This study investigated the wound healing potential of *Khaya senegalensis* leaves through a comprehensive approach, including phytochemical, antioxidant, cytotoxicity, migration, and molecular docking analyses. Bioassay-guided isolation identified β-sitosterol and stigmasterol, with structures confirmed by NMR and FTIR analyses. Phytochemical screening revealed the presence of flavonoids, saponins, triterpenoids, tannins, and steroids, with solvent polarity significantly influencing composition. The ethyl acetate extract exhibited the strongest DPPH radical scavenging (IC₅₀ = 5.6 mg/mL). The methanol extract had the highest total phenolic content (198.8 mg GAE/g) while the ethyl acetate had the highest flavonoid content (1,228.1 mg QE/g). MTT assays confirmed the cytocompatibility of all extracts, fractions, and isolated compounds in HaCaT and HDF cells (>50% viability at ≤100 µg/mL). Scratch assays demonstrated significant, concentration-dependent wound closure, with optimal effects at 12.5 μg/mL. The most active fraction (1.2H) and subfractions (1.2.1H, 1.2.3H), as well as the isolated compounds, achieved complete wound closure in HDF cells within 24 hours. Molecular docking revealed strong binding of β-sitosterol and stigmasterol with TNF-α and GSK-3β, supporting their role in modulating inflammatory and proliferative pathways. These findings highlight *Khaya senegalensis* as a promising candidate for promoting wound repair, exerting antioxidant, cytocompatible, and pro-migratory effects.

## 1. Introduction

Wound healing is a complex biological process. It restores the structure and function of damaged tissues through overlapping phases: hemostasis, inflammation, proliferation, and remodeling. These phases are controlled by intricate molecular and cellular mechanisms [1]. The pathophysiology of chronic wounds often involves a failure to progress through these normal stages, leading to a persistent inflammatory state and impaired tissue regeneration [2]. However, impaired or delayed wound healing can lead to chronic wounds, infections, and other complications, posing a significant global healthcare burden. Chronic wounds affect approximately 2% of the population in developed countries, with annual treatment costs exceeding USD 25 billion in the United States alone [3]. In resource-limited regions, the challenge is even greater due to restricted access to advanced wound care, underscoring the urgent need for effective, affordable, and accessible therapies.

In Malaysia, wound healing remains a major public health challenge, compounded by the rising prevalence of non-communicable diseases (NCDs) such as diabetes, an aging population, and disparities in healthcare access, particularly in rural areas [4]. Chronic wounds, including diabetic foot ulcers, frequently result in severe complications such as infections and amputations, further straining the healthcare system and increasing economic burdens [5]. Despite advancements in wound care technologies, inequities in resource allocation and limited access to specialized care continue to hinder effective wound management nationwide [6].

Given these challenges, natural products have emerged as a promising and cost-effective alternative for wound healing, particularly in resource-limited settings. Medicinal plants have been used for centuries in wound management due to their multifunctional properties, including antimicrobial, antioxidant, and anti-inflammatory effects [7]. These properties are crucial for wound repair, as they help reduce oxidative stress, prevent infections, and accelerate tissue regeneration. The growing recognition of these benefits has spurred ethnobotanical research as a valuable strategy for identifying novel wound healing agents [8].

Among medicinal plants with wound healing potential, *Khaya senegalensis* (Desr.) A. Juss. (African mahogany) stands out as a promising candidate. Native to sub-Saharan Africa, this tree is valued not only for its high-quality timber but also for its diverse ethnomedicinal applications, which include the treatment of wounds, fevers, and malaria [9,10]. Extensive investigations have confirmed *Khaya senegalensis* as a therapeutically important plant. It shows strong antioxidant [11], anti-inflammatory [12], and broad-spectrum antimicrobial activities against bacteria [13,14] and fungi [15]. Its other bioactivities include antihyperglycemic [16–18], hepatoprotective [19], neuroprotective [20], and anticancer [21] effects. These properties justify investigating its role in complex conditions such as impaired wound healing, especially in diabetes.

However, a large research gap exists. Pharmacological studies have mostly focused on the stem bark and seeds [9–12,14–21]. The leaves, which are more sustainable and renewable, have been overlooked. Although leaves are commonly used in traditional wound care [9], their wound-healing potential and bioactive constituents remain uncharacterized. While the antimicrobial activity of leaf extracts has been reported [13,14], no thorough, bioassay-guided studies have isolated active compounds or examined their effects on cellular wound repair mechanisms.

This study was therefore designed to address this gap by providing the first comprehensive investigation into the wound-healing properties of *Khaya senegalensis* leaves. We employed bioassay-guided fractionation, a method that combines biological activity testing with chemical separation to isolate active compounds [22]. The effects on cell migration and proliferation were evaluated using *in vitro* scratch assays and viability tests on human keratinocytes (HaCaT) and human dermal fibroblasts (HDF). Furthermore, molecular docking studies were used to explore potential interactions between phytoconstituents and key protein targets involved in wound repair.

By systematically identifying bioactive compounds from *Khaya senegalensis* leaves that promote fibroblast migration and engage critical molecular targets, this research aims to bridge the gap between traditional knowledge and modern drug discovery. The findings could support the development of affordable, evidence-based, plant-derived therapeutic agents for wound management, particularly suited for resource-constrained healthcare settings.

## 2. Materials and methods

### 2.1. Chemicals and reagents

All chemicals and solvents used in this study, including 2,2-diphenyl-1-picrylhydrazyl (DPPH), aluminium chloride, ammonia, ascorbic acid, chloroform, ethyl acetate, gallic acid, *n*-hexane, methanol, silica gel, sodium carbonate, and sulfuric acid, were purchased from Merck (Germany). Folin-Ciocalteu reagent, Mayer’s reagent, dimethyl sulfoxide (DMSO), 3-(4,5-dimethylthiazol-2-yl)-2,5-diphenyl-2H-tetrazolium bromide (MTT), and trypsin were obtained from Sigma-Aldrich (USA). Cell culture materials, such as Dulbecco’s Modified Eagle Medium (DMEM), fetal bovine serum (FBS), penicillin-streptomycin, phosphate-buffered saline (PBS), and trypan blue, were sourced from ThermoFisher Scientific (USA). Allantoin (ThermoFisher) was used as the positive control in biological assays.

### 2.2. Instrumentation

Melting points were determined using the Stuart SMP-10 apparatus (Barloworld Scientific, Staffordshire, United Kingdom). Fourier-transform infrared (FTIR) spectra were acquired on a Bruker Alpha II Compact FT-IR spectrometer (Bruker Bioscience, Billerica, MA, USA) and a Perkin Elmer Universal ATR FT-IR spectrometer (PerkinElmer, Waltham, MA, USA). Nuclear magnetic resonance (NMR) analyses (^1^H, ^13^C, COSY, HSQC, and HMBC) were performed on a Bruker Avance 500 FT-NMR spectrometer operating at 500 MHz for ^1^H and 125 MHz for ^13^C. The UV–visible absorbance spectra were obtained using a Shimadzu UV-2600 spectrophotometer, while optical density (OD) for cell viability assays was measured with a Clariostar microplate reader (BMG Labtech, Ortenberg, Germany).

### 2.3. Sample collection and plant extraction

*Khaya senegalensis* leaves were collected from Universiti Sains Malaysia (USM). A voucher specimen (USM/S Kajihayat 11834) was kept at The Herbarium, School of Biological Sciences, USM. Dried leaves were ground and sequentially macerated with *n*-hexane, ethyl acetate, and methanol. Extracts were concentrated using a rotary evaporator and stored at 4°C until further use.

### 2.4. Phytochemical screening

Qualitative phytochemical screening of the dried, powdered leaves was performed to identify the major classes of secondary metabolites present. The screening was conducted using standard qualitative chemical methods as described by [23], with slight modifications. The detailed procedures are provided in Supporting Information (S1 Table), while a summary of the tests performed is shown in Table 1.

**Table 1:** Summary of qualitative phytochemical screening of *Khaya senegalensis* leaf extracts.

Phytochemical Class	Test Name [23]	Positive Observation
Alkaloids	Mayer’s test	Formation of a cream-coloured precipitate
Flavonoids	Alkaline reagent test	Bright yellow colour that disappears upon acid addition
Saponins	Frothing test	Formation of persistent foam layer
Steroids	Salkowski test	Red coloration in upper layer and yellow-green fluorescence in lower layer
Tannins	Acetic anhydride test	Formation of green coloration
Triterpenoids	Liebermann–Burchard test	Reddish-violet colour formation

### 2.5. Antioxidant and phytochemical assays

#### 2.5.1. DPPH free radical scavenging assay.

The antioxidant activity of *Khaya senegalensis* leaf extracts was evaluated using the DPPH free radical scavenging assay, as described in [24] with minor modifications. Briefly, 0.75 mL of the samples at concentrations of 3.125 to 10 mg/mL were mixed with 1.5 mL of DPPH solution (20 μg/mL in methanol) and incubated at room temperature in the dark for 15 minutes. The absorbance was measured at 517 nm using a UV-Vis spectrophotometer. Methanol served as the control, and ascorbic acid as the positive standard. The percentage of DPPH radical scavenging activity was calculated using the following equation:


$$\text{DPPH}\;\text{radical}\;\text{scavenging}\;\text{capacity}\;(\%)\;=\;\frac{\left(A_{blank}\;-\;{A\;}_{sample}\right)}{A_{blank}}\;\;\;\;\times \;\text{1}\text{0}\text{0}\;$$ (1)

where $A_{sample}$ is the absorbance of DPPH mixed with the sample, and $A_{blank}$ is the absorbance of DPPH. All measurements were performed in triplicate and reported as the average value. The IC₅₀ value was determined from the dose-response curve provided in the Supporting Information (S1 Fig).

#### 2.5.2. Determination of total phenolic content (TPC).

The TPC of the extracts was determined using the Folin-Ciocalteu method [25] with minor modifications. Briefly, 200 µL of the sample was mixed with 1.5 mL of 10% Folin-Ciocalteu reagent and incubated in the dark for 5 minutes. Then, 1.5 mL of 5% Na₂CO₃ solution was added, and the mixture was incubated in the dark at room temperature for 2 hours. The absorbance was measured at 750 nm against a methanol blank using a UV-Vis spectrophotometer. Gallic acid standard (0.2–1.0 mg/mL, prepared in methanol) was used to generate a calibration curve. TPC was calculated as follows:


$$\text{T}\;=\;\frac{\text{C}\;\times \;\text{V}}{\text{M}}$$

where T represents total phenolic content (expressed as mg GAE/g extract), C is the concentration of gallic acid obtained from the calibration curve (mg/mL), V is the volume of extract (mL), and M is the mass of sample extract (g). All measurements were performed in triplicate.

#### 2.5.3 Determination of total flavonoid content (TFC).

The TFC was determined using an aluminum chloride colorimetric assay with quercetin as the standard [25]. Quercetin standard and the sample extracts (1 mg/mL in methanol) were prepared. 0.25 mL of the sample solution was mixed with 1.25 mL of deionized water, followed by the addition of 0.75 mL of a 5% (w/v) sodium nitrite (NaNO₂) solution. After 6 minutes, 0.15 mL of a 10% (w/v) aluminum chloride (AlCl₃) solution was added and left to react for 5 minutes. Then, 0.5 mL of 1 M sodium hydroxide (NaOH) was added, and the total volume was adjusted to 3 mL with deionized water and mixed thoroughly. The absorbance was measured immediately at 510 nm against a blank. The TFC was calculated from a quercetin calibration curve (0.2–1.0 mg/mL) using the previous equation (2):


$$\text{T}\;=\;\frac{\text{C}\;\times \;\text{V}}{\text{M}}$$ (2)

where T represents the total flavonoid content (expressed as mg QE/g extract), C is the concentration of quercetin obtained from the calibration curve (mg/mL), V is the volume of extract (mL), and M is the mass of sample extract (g). All tests were done in triplicate.

### 2.6. Bioassay-guided fractionation and isolation

The *n*-hexane extract of *Khaya senegalensis* leaves showed the highest activity in the scratch assay and was selected for further fractionation. A total of 6.93 g of this extract was separated by column chromatography using silica gel (0.015 – 0.040 mm). Elution was performed with gradients of *n*-hexane:ethyl acetate (100:0 to 40:60 v/v), followed by dichloromethane:methanol (80:20 to 100:0 v/v), producing 154 fractions. These were combined into six groups (1.1H - 1.6H) based on TLC analysis using *n*-hexane:ethyl acetate (70:30 v/v) as the mobile phase. The spots were visualized under UV light at 254 and 366 nm wavelengths and by spraying with the vanillin/H_2_SO_4_ reagent.

Biological testing with MTT and scratch assays identified fraction 1.2H as the most bioactive. This fraction underwent further purification with silica gel (0.040–0.063 mm) chromatography using *n*-hexane:ethyl acetate gradient (90:10 to 70:30 v/v) to yield six subfractions (1.2.1H-1.2.6H). After TLC and bioactivity (MTT and scratch) assays, subfraction 1.2.3H showed the greatest wound-healing activity. Repeated washing of 1.2.3H with *n*-hexane led to the isolation of a phytosterol mixture for structure analysis.

### 2.7. Structure elucidation of active compounds

The structures of isolated compounds were elucidated using IR and NMR spectroscopy. Samples were dissolved in deuterated chloroform (CDCl₃, 0.6 mL). Chemical shifts (δ) were referenced to the residual solvent peak (δ_H_ 7.26 ppm, δ_C_ 77.0 ppm) or tetramethylsilane (TMS) as the internal standard. All spectra were collected at 298 K without solvent suppression. Data processing was done using TopSpin version 3.7.0 and MestReNova version 16.0.0–39276. ^1^H and ^13^C NMR chemical shifts, multiplicities, and coupling constants are provided in Table 3, with fully integrated spectra available in the Supporting Information (S2–S5 Figs).

**Table 3:** 1H-NMR (in CDCl_3_, 500 MHz) and ^13^C-NMR (in CDCl_3_, 125 MHz) data of Compounds 1 and 2.

Position	1		2	
***δ***_**H**_ **(*J* in Hz)**	***δ*c**	***δ***_**H**_ **(*J* in Hz)**	***δ*c**
1	1.04 (m), 1.83 (m)	37.4	1.09 (m), 1.86 (m)	37.4
2	1.51 (m), 1.85 (m)	31.8	1.51 (m), 1.85 (m)	31.9
3	3.52 (m)	72.0	3.52 (m)	72.0
4	2.23 (m), 2.28 (m)	42.4	2.23 (m), 2.29 (m)	42.3
5	–	140.9	–	140.9
6	5.34 (m)	121.8	5.34 (m)	121.9
7	1.83 (m), 1.99 (m)	32.1	1.53 (m), 1.99 (m)	32.0
8	1.44 (m)	32.0	1.50 (m)	31.8
9	0.94 (m)	50.4	0.95 (m)	50.3
10	–	36.7	–	36.8
11	1.43(m), 1.51(m)	21.2	1.44 (m), 1.52 (m)	21.3
12	1.15 (m), 1.21 (m)	40.0	1.09 (m), 1.99 (m)	39.8
13	–	42.5	–	42.5
14	1.00 (s)	56.9	1.00 (s)	57.0
15	1.06 (m), 1.57 (m)	24.5	1.13 (m), 1.57 (m)	24.5
16	1.26 (m), 1.85 (m)	28.4	1.28 (m), 1.72 (m)	29.1
17	1.11 (m)	56.2	1.16 (m)	56.1
18	0.69 (s)	12.0	0.67 (s)	12.2
19	1.00 (s)	19.5	1.00 (s)	19.2
20	1.36 (m)	36.3	2.04 (m)	40.7
21	0.85 (s)	19.2	1.02 (s)	21.4
22	1.02 (m), 1.33 (m)	34.1	5.14 (dd, J = 15.1, 8.7)	138.3
23	1.16 (m), 1.19 (m)	26.3	5.00 (dd, J = 15.1, 8.7)	129.4
24	0.91 (d, J = 6.3)	46.0	1.54 (m)	51.4
25	1.66 (m)	29.3	1.84 (m)	36.7
26	1.02 (s)	20.0	0.84 (s)	19.5
27	0.85 (s)	19.0	0.81 (s)	19.0
28	1.28 (m)	23.2	1.17 (m), 1.43 (m)	25.6
29	0.85 (s)	12.2	0.69 (s)	12.2

**Note:** s = singlet, d = doublet, dd = doublet of doublet, m = multiplet. *δ*_H_ (proton chemical shifts, *J* in Hz), *δ*c (carbon chemical shifts).

### 2.8. Cell culture

HaCaT cells were purchased from AddexBio (USA), and HDF cells were obtained from the Centre for Tissue Engineering and Regenerative Medicine, Universiti Kebangsaan Malaysia (UKM). Both cell lines were maintained in Dulbecco’s Modified Eagle Medium (DMEM) with 10% fetal bovine serum (FBS) and 1% penicillin-streptomycin. Cells were incubated at 37°C in a humidified atmosphere containing 5% CO₂. Cells were cultured in 75 cm² flasks and subcultured upon reaching 70–80% confluence. For subculturing, cells were washed with phosphate-buffered saline (PBS), detached using 0.25% trypsin, and neutralized with complete medium. Cell counting was done using an automated cell counter with trypan blue staining. Samples (extracts, fractions, subfractions, and isolated compounds) were dissolved in dimethyl sulfoxide (DMSO) and diluted in DMEM to working concentrations, ensuring the final DMSO concentration was below 0.1% (v/v), which was confirmed to be non-toxic to the cells [26].

### 2.9. Biological assays

#### 2.9.1. *In vitro* cytotoxicity (MTT) assay.

The cytotoxicity of *Khaya senegalensis* extracts and compounds was assessed using the MTT assay as described by [26] with minor modifications. 100 μL of HaCaT and HDF were seeded in a 96-well plate (1 × 10^5^ cells/mL) and incubated overnight at 37°C with 5% CO_2_ until 70% confluent. After replacing the medium, cells were treated with 100 μL of positive control and samples at different concentrations (3.125, 6.25, 12.5, 25.0, 50.0, and 100.0 μg/mL) and incubated for 24 hours. The culture medium was removed from the wells, followed by the addition of 50 μL of MTT solution and 2 hours of incubation. The MTT solution was discarded and replaced with 100 μL isopropanol, shaken gently for 30 minutes on a shaker (Orbital Shaker 05–20, Boeco, Germany) at 1600 rpm. The absorbance was measured at 570 nm with a Clariostar BMG Labtech microplate reader. Cell viability was calculated according to the following equation:


$$\text{Cell}\;\text{viability}\;\%=\frac{{\text{OD}}_{\text{570a}\;\text{of}\;\text{treated}\;\text{sample}}\;}{{\text{OD}}_{\text{570b}\;\text{of}\;\text{untreated}\;\text{control}}}\times \text{1}\text{0}\text{0}$$ (3)

where ${\text{OD}}_{\text{570a}\;\text{of}\;\text{treated}\;\text{sample}}$ is the mean optical density of cells treated with the test sample, while ${\text{OD}}_{\text{570b}\;\text{of}\;\text{untreated}\;\text{control}}$ is the mean optical density of untreated control cells. The experiment was done in triplicate for each cell line. Data were expressed as a mean ± standard deviation (SD).

#### 2.9.2. *In vitro* scratch assay.

The migration and spreading capabilities of HaCaT and HDF cells were evaluated using the scratch assay method, as described by [26], using the extracts, fractions, subfractions, and isolated compounds of *Khaya senegalensis* leaves. HaCaT and HDF cells were separately seeded in 6-well plates (2.5 × 10⁵ cells/mL) and grown to 70% confluence, forming a monolayer. A linear scratch was made across the cell layer using a sterile yellow pipette tip, followed by washing with PBS to remove debris. 2 mL of fresh DMEM containing test samples (12.5–50 μg/mL) was added. DMEM alone served as the negative control, and DMEM supplemented with allantoin was used as the positive control. The plates were incubated at 37°C with 5% CO₂. Images of the wounded area were taken at specific time points (HDF cells: 0, 13, 15, 18, 21, and 24 hours; HaCaT cells: 0, 12, 24, 36, and 48 hours), and analyzed using ImageJ software to measure wound closure. All tests were performed in triplicate, and data were expressed as mean ± SD. The percentage of wound closure was calculated using the following formula:


$$\text{Wound}\;\text{closure}\;\%\;=\;\frac{\text{Wound}\;\text{area}\;\text{before}\;\text{treatment}-\text{Wound}\;\text{area}\;\text{after}\;\text{treatment}}{\text{Wound}\;\text{are}\;\text{before}\;\text{treatment}}\;\times \;\text{1}\text{0}\text{0}\%$$ (4)

### 2.10. Molecular docking studies

Molecular docking simulations were performed using AutoDock Tools version 1.5.7 and BIOVIA Discovery Studio v21.1.0.20298 to study interactions between *Khaya senegalensis* isolated compounds and key therapeutic targets. Three targets were selected based on their established roles: Tumor Necrosis Factor-alpha (TNF-α), a primary mediator of the inflammatory phase; Glycogen Synthase Kinase-3β (GSK-3β), a regulator of inflammation and cellular proliferation; and Transforming Growth Factor-beta 1 (TGF-β1), a master regulator of fibroblast activation and tissue remodeling [27,28]. The crystal structures of TNF-α (PDB ID: 2AZ5), TGF-β1 (PDB ID: 6B8Y), and GSK-3β (PDB ID: 5K5N) were retrieved from the Protein Data Bank (https://www.rcsb.org). The compounds were drawn using ChemDraw version 23.1.2.7.

Proteins were prepared in Discovery Studio by removing water molecules, adding hydrogen atoms, and defining the catalytic binding sites based on native ligand coordinates. A 10 × 10 × 10 Å grid box was established around each binding site for docking. Proteins and ligands were then converted into PDBQT format using AutoDock Tools version 1.5.7, with Kollman charges applied to account for electrostatic interactions, while Gasteiger charges and rotatable bonds are defined for ligands. Docking simulations were conducted using AutoDock Vina using an exhaustiveness value of 8 to ensure sufficient conformational sampling. Each docking was run in triplicate to ensure reproducibility and consistency. Binding poses were visualized and analyzed using BIOVIA Discovery Studio Visualizer. Binding affinities (in kcal/mol), hydrogen bonding interactions, hydrophobic contacts, and other non-covalent interactions were evaluated to assess ligand binding behavior.

### 2.11. Statistical analysis

Data are expressed as mean ± SD from at least three independent experiments (n = 3). Statistical differences between groups were analyzed using one-way analysis of variance (ANOVA), followed by Tukey’s post hoc test for multiple comparisons (SPSS Statistics 21, IBM). Results with *p*-values less than 0.05 (*p* < 0.05) were considered statistically significant.

## 3. Results and discussions

### 3.1. Phytochemical screening

The phytochemical analysis of *Khaya senegalensis* leaf extracts revealed distinct classes of bioactive compounds depending on solvent polarity (Table 2). The *n*-hexane extract (non-polar) predominantly contained flavonoids, triterpenoids, saponins, and steroids [29]. The ethyl acetate extract (moderately polar) demonstrated the presence of flavonoids, saponins, steroids, tannins, and triterpenoids. In contrast, the methanol extract (polar) exhibited a broader range of compounds, including flavonoids, saponins, steroids, tannins, and triterpenoids, which can be attributed to methanol’s strong ability to extract polar phytochemicals [30,31]. Overall, this solvent-based extraction approach effectively captured a wide spectrum of phytochemicals from *Khaya senegalensis*, supporting its application as a preparative step for identifying potential bioactive constituents.

**Table 2:** Qualitative phytochemical profile of *Khaya senegalensis* leaves extracts obtained using solvents of varying polarity.

Phytochemical test	*n*-hexane extract	Ethyl acetate extract	Methanol extract
**Alkaloids**	x	x	x
**Flavonoids**	+	+	+
**Saponins**	+	+	+
**Steroids**	+	+	+
**Tannins**	x	+	+
**Triterpenoids**	+	+	+

**Note:** “+” indicates presence; “x” indicates absence.

### 3.2. DPPH free radical scavenging assay

The DPPH radical scavenging activity was evaluated based on the percentage inhibition of free radicals by antioxidant compounds present in the extracts. The IC₅₀ values were determined from linear regression of dose-response curves (3.125–10 mg/mL), as provided in Supporting Information (S1 Fig). A lower IC₅₀ value indicates stronger antioxidant activity, as it reflects the concentration required to scavenge 50% of the free radicals [32]. The antioxidant capacity of each extract depends on both the abundance and the free radical-neutralizing compounds.

**Fig 1:**
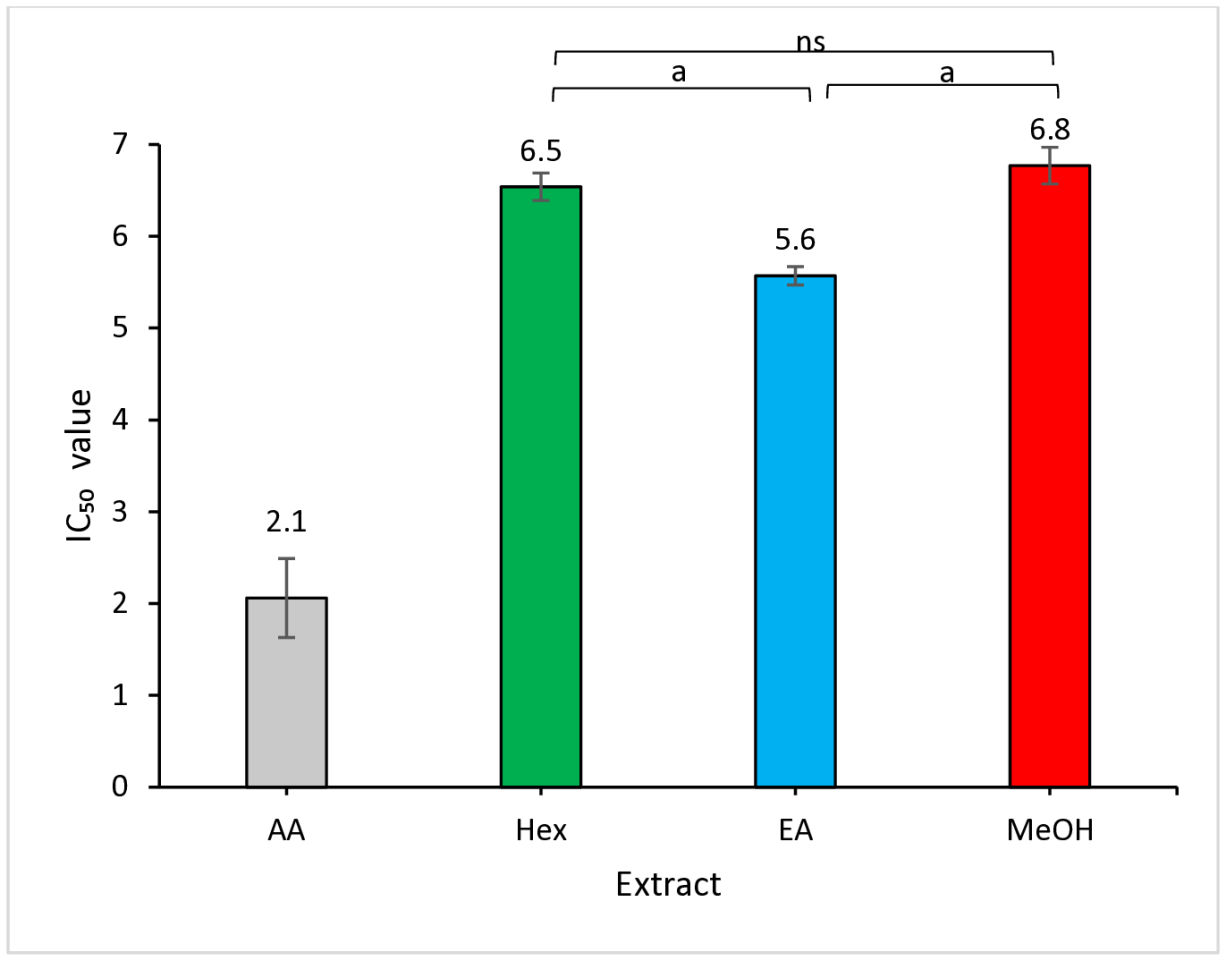
Antioxidant capacity of *Khaya senegalensis* leaf extracts determined by the DPPH free radical scavenging assay. Results are expressed as mean ± SD from three independent experiments; error bars represent SD. Data were analyzed using one-way ANOVA followed by Tukey’s post hoc test. Significant differences between groups are indicated by alphabet and ‘ns’ (^a^*p* < 0.001; ns = not significant). All extracts were significantly less potent than standard ascorbic acid (AA) at ^a^*p* < 0.001 (not shown for clarity). Hex = *n*-hexane extract; EA = ethyl acetate extract; MeOH = methanol extract.

In this study, *Khaya senegalensis* extracts demonstrated concentration-dependent radical scavenging activity, with IC₅₀ values below 10.0 mg/mL, as shown in Fig 1. Among the tested extracts, the ethyl acetate fraction exhibited the strongest activity (IC₅₀ = 5.6 ± 0.5 mg/mL), followed by *n*-hexane (IC₅₀ = 6.5 ± 0.8 mg/mL) and methanol (IC₅₀ = 6.8 ± 1.1 mg/mL). Statistical analysis revealed that all crude extracts were significantly less potent than the standard ascorbic acid (IC₅₀ = 2.1 ± 2.2 mg/mL; *p* < 0.001). However, comparisons among the extracts revealed that the superior activity of the ethyl acetate fraction was significant compared to both the *n*-hexane and methanol extracts (*p* < 0.001). This finding suggests that the most active antioxidant compounds in this plant are of medium polarity. No significant difference was observed between the *n*-hexane and methanol extracts (*p* > 0.05).

These solvent-dependent trends are consistent with previous studies. For instance, Assefa et al. [33] showed that ethyl acetate extracts of citrus peels (yuzu, hallabong, and orange) displayed stronger DPPH radical scavenging activity (IC₅₀ = 0.37, 0.12, and 0.04 mg/mL, respectively) than methanol and *n*-hexane extracts. The superior antioxidant activity of the ethyl acetate extract (IC₅₀ = 5.6 mg/mL) aligns with findings from Ariffin et al. [34], who also reported ethyl acetate as the most effective solvent for extracting antioxidants from *Laurus nobilis* leaves. Taken together, these findings suggest that ethyl acetate is more effective than methanol for extracting antioxidant compounds from plant materials [35]. This difference is primarily attributed to solvent polarity, which influences the types of phytochemicals that can be extracted. Ethyl acetate’s intermediate polarity makes it particularly efficient at isolating flavonoids and phenolic compounds, which possess strong radical-scavenging capacity due to their hydroxyl groups [30,36]. This explains the superior antioxidant performance of ethyl acetate fractions in the DPPH assay in the present study.

### 3.3. Determination of total phenolic content (TPC) and total flavonoid content (TFC)

TPC and TFC were quantified using standardized spectrophotometric assays, widely regarded as the gold standard for preliminary phytochemical screening [37]. TPC is determined through the Folin–Ciocalteu reduction method, which measures hydroxyl-group-containing compounds, while TFC is measured using aluminum chloride colorimetry, which detects flavonoid conjugates. Results are expressed as gallic acid equivalents (GAE) and quercetin equivalents (QE) per gram of extract, respectively [38].

**Fig 2:**
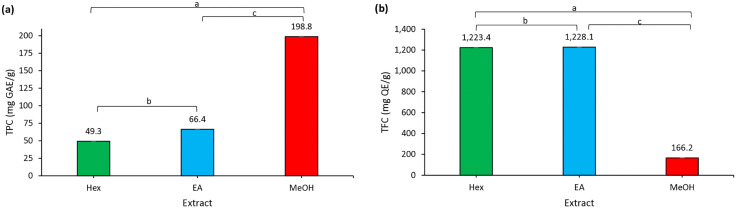
(a) Total phenolic content (TPC) and (b) total flavonoid content (TFC) in *Khaya senegalensis* leaf extracts. Results are expressed as mean ± SD from three independent experiments; error bars represent SD. Data were analyzed using one-way ANOVA followed by Tukey’s post hoc test for multiple comparisons between all extract groups. Significant differences between specific groups are indicated by brackets with alphabets: ^a^*p* < 0.001, ^b^*p* < 0.01, ^c^*p* < 0.05. Hex = *n*-hexane extract; EA = ethyl acetate extract; MeOH = methanol extract.

As shown in Fig 2 (a), the methanol extract exhibited a significantly higher TPC (198.8 ± 0.4 mg GAE/g) than both the ethyl acetate (66.4 ± 0.0 mg GAE/g, *p* < 0.05) and *n-*hexane (49.3 ± 0.1 mg GAE/g, *p* < 0.001) extracts. The ethyl acetate extract also had a significantly higher TPC than the *n-*hexane extract (*p* < 0.01). Analysis of the TFC in Fig 2 (b) revealed that both the ethyl acetate (1,228.1 ± 0.1 mg QE/g) and *n-*hexane (1,223.4 ± 0.0 mg QE/g) extracts demonstrated significantly higher levels than the MeOH extract (166.2 ± 0.0 mg QE/g, *p* < 0.001). Furthermore, the ethyl acetate extract was significantly higher than the *n-*hexane extract (*p* < 0.01). These results demonstrate that solvent polarity plays a decisive role in the extraction of bioactive compounds from *Khaya senegalensis*. Methanol, being highly polar, efficiently extracts phenolic compounds [38], ethyl acetate, with its intermediate polarity, is more effective for flavonoids [39]. This polarity-dependent selectivity highlights the importance of solvent choice when targeting specific phytochemical classes. Sequential extraction using solvents of increasing polarity has been recommended to comprehensively capture the full phytochemical profile of medicinal plants [40,41].

In this study, the consistently higher flavonoid content in ethyl acetate aligns with previous reports. Assefa et al. [33] reported phenolic and flavonoid contents of citrus peel extracts ranging from 58.2 ± 1.4 to 102.4 ± 8.6 mg/g and 19.6 ± 0.5 to 64.3 ± 0.8 mg/g dry extract, respectively, with ethyl acetate extracts outperforming methanol and *n*-hexane. Ariffin et al. [34] found that ethyl acetate extracts of *Laurus nobilis* leaves contained significantly higher flavonoid (0.0628 mg QE/g) and phenolic contents (0.0198 mg GAE/g) compared to other solvents. However, our study found a dramatically higher TFC in *Khaya senegalensis* (1,228.1 mg QE/g) compared to their report (0.0628 mg QE/g), highlighting the remarkable flavonoid richness of this plant species and its potential as a superior source of natural antioxidants.

### 3.4. Structure elucidation of active compounds

Compound **1** was observed as a white waxy powder with a melting point of 134–136°C. IR spectral analysis reveals a broad absorption at 3386 cm⁻¹ corresponding to O–H stretching, alongside peaks at 2936 cm⁻¹ (C–H stretching of CH₂), and 2868 cm ⁻ ¹ (C–H stretching of CH groups). A band at 1464 cm⁻¹ indicates C = C stretching from the sterol nucleus, while the absorption at 1061 cm⁻¹ corresponds to C–O stretching of the hydroxyl group. The absence of a band near 965 cm⁻¹ confirms the lack of a trans-disubstituted double bond in the side chain [42].

The ¹H NMR spectrum showed the typical signals of a sterol nucleus, including an olefinic proton at δ_H_ 5.34 (m, H-6) and an oxygenated methine proton at δ_H_ 3.52 (m, H-3). Six methyl signals appeared between δ_H_ 0.69 and 1.02, assigned to H-18, H-19, H-26, H-27, and H-29, consistent with the sterol framework. Importantly, no olefinic side-chain protons were observed. The ^13^C NMR including olefinic carbons at δ_C_ 140.9 (C-5) and 121.8 (C-6), an oxygenated methine at δ_C_ 72.0 (C-3), and aliphatic carbons at δ_C_ 34.1 (C-22) and 26.3 (C-23), indicative of a saturated side chain. The ¹H and ¹³C NMR data for compound **1** are presented in Table 3. DEPT-135 spectra confirmed 29 carbon atoms consist of six methyl, eleven methylene, nine methane, and three quartenary carbon. Evidence from 2-D NMR strengthened this assignment. The ^1^H–^1^H COSY spectrum showed correlations within the sterol skeleton of H-2 to H-3/H4, H-6 to H-7, H-24 to H-25, and H-25 to H-27 while the HMBC spectrum confirmed key connectivities, including H-4 correlations to C-1, C-3, C-5, and C-6. Collectively, the data confirmed the structure as β-sitosterol [43]. Fully integrated spectra are provided in the Supporting Information (S2–S3 Figs).

Compound **2** was observed as anhydrous white powder, with a melting point of 165–168°C. The IR spectrum shows a broad O–H stretching band at 3323 cm⁻¹, along with C–H stretching absorptions at 2936 cm⁻¹ and 1371 cm⁻¹. A C = C stretching band is observed at 1664 cm⁻¹, with an additional band at 1456 cm⁻¹ assigned to skeletal vibrations of the sterol ring. Notably, the absorption at 965 cm⁻¹ is characteristic of a trans-disubstituted double bond in the side chain, distinguishing stigmasterol from β-sitosterol. The band at 1055 cm⁻¹ further confirms C–O stretching from the hydroxyl group [44].

The ¹H NMR spectrum resembled that of β-sitosterol in showing the sterol nucleus signals at δ_H_ 5.34 (H-6) and δ_H_ 3.52 (H-3). The key difference was the presence of two olefinic protons in the side chain, observed at δ_H_ 5.14 (dd, J = 15.1, 8.7 Hz, H-22) and δ_H_ 5.00 (dd, J = 15.1, 8.7 Hz, H-23). The ^13^C NMR further confirmed unsaturation in the side chain, with signals at δ_C_ 138.3 (C-22) and 129.4 (C-23), replacing the aliphatic carbons found in β-sitosterol. The ¹H and ¹³C NMR data for compound **2** are presented in Table 3. The DEPT-135 spectra confirmed twenty-nine carbon atoms, consisting of six methyl, nine methane, eleven methylene, and three quaternary carbons. The structure was further substantiated by 2-D NMR data. The ^1^H–^1^H COSY spectrum clearly showed the spin system of the unsaturated side chain, with correlations between H-3 to H-2/H-4, H-6 to H-7, H-11 to H-12, H-20 to H-21/H-22, and H-23 to H-22/H-24. The HMBC spectrum provided long-range correlations that confirmed the position of the double bond, including H-22 to C-23 and H-23 to C-22, while correlations from H-6 to C-4/ C-7/ C-10 established the connectivity of the unsaturated side chain to the core. These findings definitively confirmed the compound as stigmasterol [43,44]. Fully integrated spectra are provided in the Supporting Information (S4–S5 Figs).

**Fig 3:**
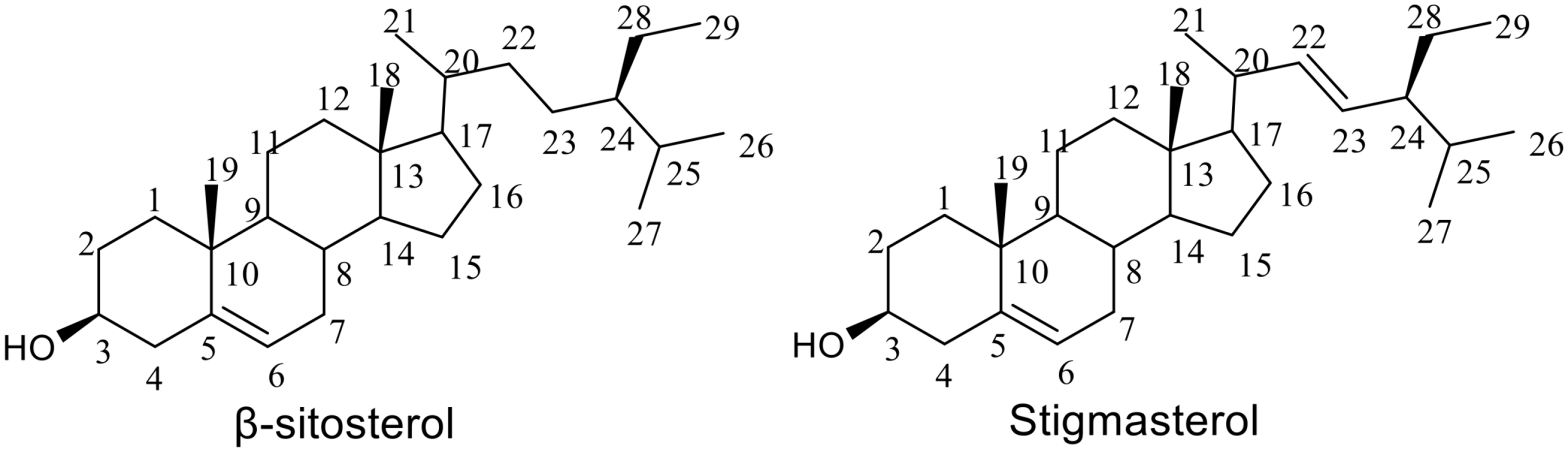
Chemical structure of the isolated compounds from *Khaya senegalensis* leaves.

In summary, the spectroscopic evidence for both compounds were in full agreement with reported values for β-sitosterol and stigmasterol [45–48]. The primary structural difference was clearly observed in the side chain, which β-sitosterol possessed a saturated ethyl side chain, while stigmasterol contained a Δ²² double bond, a distinction that was confirmed through IR and NMR analyses. The result of a positive steroid test further supports the isolation of sterol types of β-sitosterol and stigmasterol. The structures of the isolated compounds are shown in Fig 3.

### 3.5. *In vitro* cytotoxicity (MTT) assay

**Fig 4:**
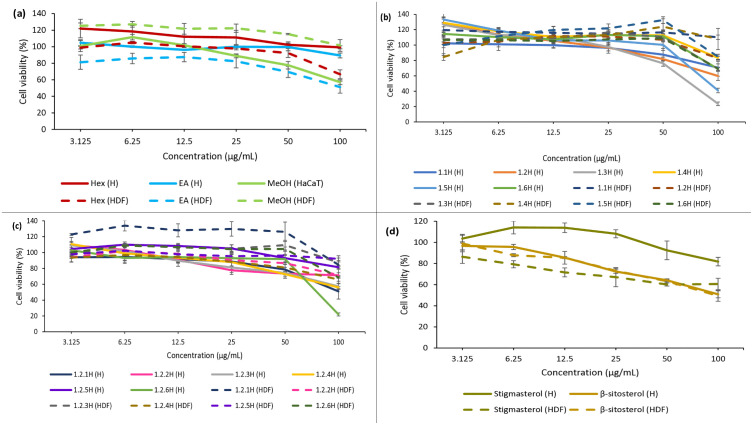
Cytotoxicity assessment of *Khaya senegalensis* derivatives. Cell viability of (a) crude extracts, (b) fractions, (c) subfractions, and (d) isolated compounds on HaCaT and HDF cells was determined by MTT assay after 24 h treatment. Results are expressed as mean ± SD from three independent experiments; error bars represent SD. Hex = *n-*hexane extract; EA = ethyl acetate extract; MeOH = methanol extract, (H) = HaCaT, (HDF) = human dermal fibroblasts.

To determine the optimal concentrations for cell culture experiments, the effects of *Khaya senegalensis* extracts on cell viability were evaluated after 24 hours of incubation using the MTT assay [49]. As shown in Fig 4 (a) to (d), exposure of both cell types to concentrations ranging from 3.125 to 100 μg/mL did not cause notable cytotoxicity, as cell viability remained above 50%. A slight decrease in cell viability was observed at higher concentrations. Nonetheless, all concentrations remained within the safe dosage range for further biological experiments.

Fig 4 (a) demonstrates that all three extracts were non-toxic to both HaCaT and HDF cells. HaCaT cell viability ranged from 100.9 ± 6.8% to 57.2 ± 2.8%, while HDF cell viability ranged from 125.2 ± 3.5% to 51.3 ± 7.2% across the extracts. Notably, the *n*-hexane extract showed high HaCaT viability (99.3 ± 1.8%) even at the highest concentration (100 µg/mL) (S2 Table). For the fractions and subfractions (S3 and S4 Tables, respectively), cell viability decreased with increasing concentrations, as illustrated in Fig 4 (b) and (c), respectively. HaCaT cell viability ranged from 133.2 ± 3.5% to 23.7 ± 2.3%, while HDF viability ranged from 119.6 ± 7.8% to 69.3 ± 9.8%. Fig 4 (d) shows that both isolated compounds exhibited high viability in HaCaT cells, ranging from 103.5 ± 4.1% to 50.9 ± 3.3%, compared to HDF cells, which showed viability between 98.9 ± 7.8% to 49.5 ± 5.2% (S5 Table).

The MTT assay results demonstrate a clear relationship with wound healing potential, as cell viability is fundamental for tissue repair processes. Our study revealed that *Khaya senegalensis* derivatives, including crude extracts, fractions, subfractions, and isolated compounds (β-sitosterol and stigmasterol), all exhibited excellent cytocompatibility in both HaCaT and HDF cells, maintaining viability above 50% at concentrations up to 100 μg/mL. Although some fractions displayed concentration-dependent decreases in viability, all remained within safe thresholds.

These findings demonstrate that all *Khaya senegalensis* derivatives, including crude extracts, fractions, subfractions, and isolated phytosterols, are cytocompatible with both keratinocytes and fibroblasts at concentrations relevant for wound-healing applications. The maintained viability is notable, as cell survival is essential for re-epithelialization, extracellular matrix (ECM) remodeling, and tissue repair. Our results are consistent with previous studies reporting high cell viability of plant-derived extracts across similar solvent systems. For example, the ethyl acetate fraction of *Moringa oleifera* was shown to be non-cytotoxic to normal human dermal fibroblasts, maintaining >90% viability and supporting wound closure assays [50]. Likewise, a methanolic fraction of *Centella asiatica* preserved fibroblast and keratinocyte viability above 80–90% at 6.25–25 μg/mL and stimulated fibroblast proliferation [51].

Similarly, a hydroethanolic extract of *Sargassum polycystum* maintained >90% viability in both fibroblasts (L929) and keratinocytes (HaCaT) up to 200 μg/mL while enhancing cell migration [52], and an ethyl acetate extract of *Caulerpa microphysa* was reported to be non-cytotoxic to fibroblasts, promoting collagen synthesis and reducing proinflammatory cytokines [53]. Together, these findings highlight that plant-derived extracts prepared in ethyl acetate, methanol, or hydroethanol are generally safe for skin cells, reinforcing that *Khaya senegalensis* extracts are well tolerated and suitable for wound-healing interventions.

### 3.6. *In vitro* scratch assay

**Fig 5:**
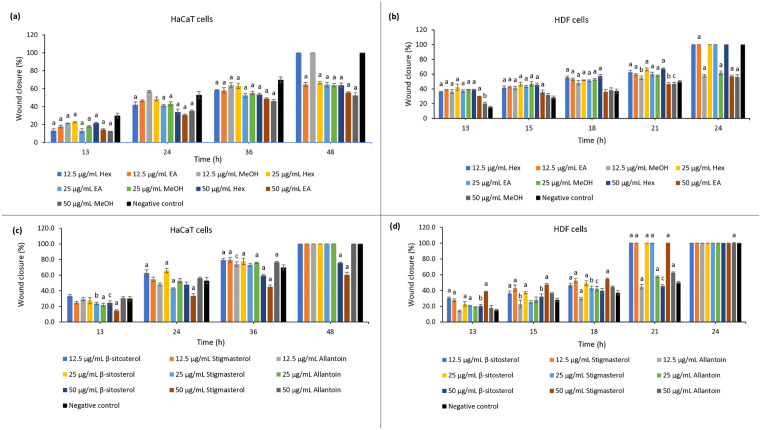
Migratory effects of Khaya senegalensis extracts and compounds. Wound closure (%) over time in (a) HaCaT cells treated with crude extracts, (b) HDF cells treated with crude extracts, (c) HaCaT cells treated with isolated compounds, and (d) HDF cells treated with isolated compounds, as measured by the scratch assay. All treatments were compared to the negative (untreated) control. Results are expressed as mean ± SD from three independent experiments; error bars represent SD. Data were analyzed using one-way ANOVA followed by Tukey’s post hoc test. ^a^*p* < 0.001, ^b^*p* < 0.01, ^c^*p* < 0.05 compared with negative control. Hex = *n*-hexane extract; EA = ethyl acetate extract; MeOH = methanol extract.

The migratory effects of *Khaya senegalensis* extracts and isolated compounds on HaCaT keratinocytes and HDF fibroblasts were evaluated using a scratch assay, an *in vitro* model that effectively simulates cell migration and proliferation during tissue repair [54], as shown in Fig 5 (a-d). All statistical analyses compared treatment groups to the negative (untreated) control to establish a baseline migratory rate of the cells. All crude extracts at concentrations of 12.5, 25, and 50 μg/mL promoted over 50% wound closure within 48 hours in HaCaT cells and within 24 hours in HDF cells (S6 Table).

Fig 5 (a), shows that both *n*-hexane and methanol extracts at 12.5 μg/mL induced complete (100%) wound closure within 48 hours in HaCaT cells. Nearly all extracts showed a highly significant cell migration (*p* < 0.001) across concentrations and time points, except for the 50 μg/mL methanol extract, which exhibited lower wound closure (52.4 ± 3.2%, *p* < 0.05). The wound healing response in HDF cells, as shown in Fig 5 (b), was more rapid. The *n*-hexane extract at all tested concentrations and the ethyl acetate extract at 12.5 and 25 μg/mL achieved complete wound closure within 24 hours. While both *n-*hexane and ethyl acetate extracts consistently showed strong significance (*p* < 0.001), the methanol extract showed variable significance over time (*p* < 0.01 at 13h; *p* < 0.05 at 21h; *p* < 0.001 at 24h).

Fractionation of the active *n-*hexane crude extract successfully identified highly potent fractions and subfractions (see S7 and S8 Tables). The most effective fraction, 1.2H, achieved complete wound closure within 24 hours in HDF cells and within 48 hours in HaCaT cells across all tested concentrations (12.5–50 µg/mL). Among the subfractions, 1.2.1H, 1.2.3H, and 1.2.4H were particularly promising, inducing 100% wound closure in HaCaT cells at specific concentrations. In HDF cells, the wound healing response was exceptionally rapid, with all subfractions except 1.2.5H and 1.2.6H achieving complete closure within 24 hours, even at the lowest concentration of 12.5 µg/mL.

Meanwhile, the isolated compounds β-sitosterol and stigmasterol exhibited potent, concentration-dependent activity as illustrated in Fig 5 (c) and (d). At 12.5 and 25 μg/mL, both compounds stimulated 100% wound closure within 48 hours in HaCaT cells and within 24 hours in HDF cells, demonstrating highly significant effects (*p* < 0.001) (S9 Table). However, at 50 μg/mL, their efficacy decreased. In HaCaT cells, β-sitosterol and stigmasterol achieved only 75.6 ± 1.0% and 60.5 ± 3.3% closure by 48 hours, respectively, with β-sitosterol showing reduced significance (*p* < 0.05 only at 13h) (S6 Fig). Similarly, in HDF cells, β-sitosterol at 50 μg/mL achieved only 45.5% closure by 21 hours with diminished significance (*p* < 0.01 at early time points), whereas stigmasterol maintained complete closure and high significance (*p* < 0.001) (S8 Fig). Visual comparison with the controls (S7 and S9 Figs) confirmed the superior wound closure promoted by the compounds at optimal concentrations.

Our findings demonstrate that *Khaya senegalensis* extracts and isolated compounds possess strong wound healing potential, with optimal effects observed at lower concentrations (12.5–25 μg/mL) that maintain cell viability. The *n*-hexane and ethyl acetate extracts, along with fraction 1.2H and its subfractions 1.2.1H and 1.2.3H, were particularly effective in promoting wound closure. Higher concentrations (50 μg/mL) showed reduced efficacy, particularly for β-sitosterol, likely due to cytotoxic effects. Notably, all tested compounds outperformed the allantoin control. Additionally, HDF cells consistently showed faster wound closure compared to HaCaT cells, highlighting the importance of cell type-specific responses and careful concentration optimization for therapeutic applications.

The efficacy of the isolated compounds β-sitosterol and stigmasterol is consistent with their established roles in wound healing. Both phytosterols have been implicated in wound healing through their anti-inflammatory and tissue repair activities. β-Sitosterol has been shown to exert notable anti-inflammatory effects in rodent models, thereby reducing inflammatory mediators that delay wound closure [55]. Stigmasterol, extensively reviewed for its pharmacological activities, also demonstrates anti-inflammatory and wound healing potential, with evidence of its role in promoting epithelialization and fibroblast proliferation [56]. Our finding that stigmasterol promotes rapid fibroblast migration corroborates a study on *Jatropha tanjorensis*, where stigmasterol-rich extracts accelerated wound contraction *in vivo* [57]. Our research provides a significant advancement by demonstrating the efficacy of the pure compounds in human cell lines, thereby deconvoluting the activity of the complex crude extract mixture. Furthermore, while β-sitosterol has been noted for its anti-inflammatory effects [55], our study is the first to computationally link its wound-healing potential to the specific inhibition of the GSK-3β and TNF-α pathways.

### 3.7. Molecular docking studies

Molecular docking analysis revealed that both β-sitosterol and stigmasterol bind stably to key wound healing targets. Stigmasterol exhibited stronger binding affinity for TNF-α (2AZ5; −7.5 ± 0.3 kcal/mol) and GSK-3β (5K5N; −7.4 ± 0.2 kcal/mol), whereas β-sitosterol showed superior binding to TGF-β1 (6B8Y; −4.9 ± 0.1 kcal/mol) (Table 4). The potent interactions with the pro-inflammatory regulator TNF-α and the proliferative mediator GSK-3β provide a plausible molecular explanation for the significant pro-migratory effects observed in the scratch assay. The stronger binding of stigmasterol to TNF-α and GSK-3β may be attributed to the additional double bond in its side chain, which enhances conformational flexibility and enables extensive hydrophobic, π-alkyl, and alkyl interactions with key residues, including Tyr59, Tyr119, Val70, Cys199, and Ile62. Notably, the strong π–σ and π–alkyl interactions with aromatic residues within the TNF-α binding pocket suggest a more stable orientation for stigmasterol.

**Table 4:** Binding affinities (kcal/mol) and protein–ligand interactions of isolated compounds from *Khaya senegalensis* leaves with selected target proteins. Results are expressed as mean ± SD from three independent experiments.

Protein	Ligands	Free energy of binding (kcal/mol)	Convectional Hydrogen bond	Residual amino acid interactions/ hydrophobic/Pi-anion/Pi-cation/Pi-alkyl/Pi-sulfur/other interactions	van der Waals interaction
**2AZ5**	Stigmasterol	−7.5 ± 0.3		π-σ Tyr59 (3.61493); π-alkyl Tyr59 (5.3145), Tyr119 (5.15743)	Leu55(D), Gly121(B), Tyr119(B), Leu120(B), Tyr151(B), Gln61(B), Gly121(A), Ile155(B), Gly122(A), Leu57(A), Leu57(B)
	β-sitosterol	−7.2 ± 0.2	Gly121 (1.70414)	π-σ Tyr119 (3.98559), π-alkyl Tyr119 (5.46846, 5.47241)	Tyr151, Gln61, Leu120, Tyr59, Gly122, Tyr151(B), Tyr59(B), Tyr119(B), Leu157(D), Leu55(D)
**5K5N**	Stigmasterol	−7.4 ± 0.2		Alkyl Val70 (5.14816), Cys199 (3.71071), Ile62 (4.53252)	Pro136, Glu137, Arg141, Thr138, Gln185, Asn186, Asp200, Phe67, Lys85, Ala83, Leu132, Val110, Leu188, Gly63, Tyr134,
	β-sitosterol	−7.3 ± 0.3	Asp133 (2.27838), Val135 (2.21778)	π-alkyl Tyr134 (5.33398), Tyr140 (5.4444); Alkyl Val70 (4.01702, 4.6063), Ala83 (3.41467, 4.74212), Leu188 (4.47062, 5.03094), Cys199 (4.71416), Arg141 (4.07003)	Gln185(A), Ser261(B), Asn64(A), Phe67(A), Asp200(A), Leu132(A), Val110(A), Ile62(A), Gly63(A), Thr138(A)
**6B8Y**	Stigmasterol	−3.9 ± 0.0	Val279 (2.54514)	Alkyl Ile211 (5.08885), Val219 (3.91804), Lys232 (4.78822), Leu260 (5.20777), Leu278 (5.35466), Ala350 (4.22733)	Ser280, Leu340, Gly286, Ser287, Phe262, Ile263, Tyr249, Glu245, Asp351, Asn338, Lys337, Gly214
	β-sitosterol	−4.9 ± 0.1		Alkyl Ile211 (4.87238), Val219 (4.78832, 5.35559), Lys232 (4.75385, 4.98123), Leu260 (4.94183), Leu340 (4.92184), Ala350 (4.35342, 4.5187), Leu260 (5.48624), Leu278 (5.22457)	His283, Gly286, Tyr282, Ser287, Ala230, Ser280, Ala264, Val279, Lys337, Asn338, Asp351, Glu245, Tyr249, Phe262

**Note:** π-alkyl = pi-alkyl; π-σ = pi-sigma

In contrast, β-sitosterol stabilized its binding to GSK-3β through hydrogen bonds with Asp133 and Val135, compensating for its relatively lower hydrophobic contributions compared to stigmasterol. For TGF-β1, the stronger binding of β-sitosterol was driven by multiple alkyl interactions with residues such as Ile211, Val219, Lys232, Leu260, Leu278, and Ala350. The single hydrogen bond formed by stigmasterol with Val279 was insufficient to offset its weaker hydrophobic engagement, indicating that the TGF-β1 binding pocket is more complementary to β-sitosterol’s sterol framework. Detailed representations of these protein-ligand interactions are provided in S10 Table.

From a biological perspective, these findings align with the established roles of these targets. TGF-β1 is a pivotal regulator of cutaneous wound repair, orchestrating fibroblast activation, myofibroblast differentiation, extracellular matrix deposition, and granulation tissue formation, while also promoting wound contraction. However, an excessive or dysregulated expression of TGF-β1 can lead to chronic wounds or fibrosis [58,59]. Specifically, the strong inhibition of TNF-α, a master regulator of inflammation, suggests that these phytosterols may accelerate the wound healing process by mitigating the prolonged inflammatory phase characteristic of chronic wounds. This is crucial, as excessive TNF-α is known to impede keratinocyte and fibroblast migration [60]. Simultaneously, their high affinity for GSK-3β points to a potential activation of the Wnt/β-catenin signaling pathway. Inhibition of GSK-3β leads to β-catenin stabilization and nuclear translocation, a key driver of cell proliferation and migration during wound re-epithelialization [61,62].

This study is the first to report the binding of β-sitosterol and stigmasterol to TGF-β1, TNF-α, and GSK-3β in the context of wound healing. These molecular targets are interconnected in wound healing, with TNF-α and GSK-3β primarily involved in the inflammatory and proliferative phases, while TGF-β1 regulates tissue remodeling and fibrotic outcomes. The selective and stronger interactions of β-sitosterol and stigmasterol with TNF-α and GSK-3β suggest that these phytosterols may exert potent anti-inflammatory and pro-repair effects without excessively activating fibrotic pathways. Therefore, the synergistic *in silico* targeting of both inflammatory (TNF-α) and proliferative (GSK-3β) pathways provides a compelling mechanistic model whereby β-sitosterol and stigmasterol likely promote wound closure by concurrently resolving inflammation and directly stimulating cellular migration programs. This dual-target mechanism aligns perfectly with the significant wound closure observed in our HaCaT and HDF scratch assays.

## 4. Conclusion

β-Sitosterol and stigmasterol were successfully isolated from *Khaya senegalensis* leaves and identified as the main bioactive compounds contributing to wound healing. Both sterols demonstrated significant cytocompatibility and enhanced the migration of fibroblasts and keratinocytes. Molecular docking revealed favourable interactions with key protein targets (TNF-α, GSK-3β, and TGF-β1), supporting a mechanistic role in modulating inflammation and tissue regeneration. Although the final isolates were non-phenolic, initial TPC and TFC analysis provided valuable guidance for extract prioritization and solvent selection, explaining the bioactivity differences between crude extracts and isolated sterols. The integrated approach of phytochemical profiling, bioassay-guided fractionation, *in vitro* evaluation, and molecular docking confirms the wound healing potential of *Khaya senegalensis*. These findings provide scientific validation for its traditional use and position β-sitosterol and stigmasterol as promising natural agents for developing plant-based wound therapies. A primary limitation of this study is its reliance on *in vitro* and computational models. Therefore, future research should focus on *in vivo* validation using animal wound models to experimentally confirm the proposed anti-inflammatory and pro-proliferative mechanisms, alongside the development of topical formulations to translate these findings into practical therapeutic applications.

## Supporting information

S1 Table Detailed procedures for qualitative phytochemical screening of *Khaya senegalensis* leaf extracts. (TIF)

S1 Fig Dose–response curves of *Khaya senegalensis* leaf extracts and standard ascorbic acid in the DPPH free radical scavenging assay. The scavenging activity (%) was plotted against the sample concentration (mg/mL). The IC₅₀ values for each sample were determined from the linear regression equations of the respective curves. Results are expressed as mean ± SD from three independent experiments; error bars indicate SD. Hex = *n*-hexane extract; EA = ethyl acetate extract; MeOH = methanol extract, AA = ascorbic acid.
(TIF)

S2 Fig ^1^H-NMR (500 MHz, CDCl3) spectrum of β-sitosterol. (TIF)

S3 Fig ^13^C-NMR (125 MHz, CDCl_3_) and DEPT-135 spectrum of β-sitosterol. (TIF)

S4 Fig ^1^H-NMR (500 MHz, CDCl_3_) spectrum of Stigmasterol. (TIF)

S5 Fig ^13^C-NMR (125 MHz, CDCl_3_) and DEPT-135 spectrum of Stigmasterol. (TIF)

S2 Table Cell viability (%) of *Khaya senegalensis* extracts in HaCaT and HDF cells. Cell viability was assessed after 24 hours of incubation via the MTT assay, with extract concentrations ranging from 3.125 to 100 μg/mL. Results are expressed as mean ± SD from three independent experiments. Hex = *n*-hexane extract; EA = ethyl acetate extract; MeOH = methanol extract.
(TIF)

S3 Table Cell viability (%) of *Khaya senegalensis* fractions in HaCaT and HDF cells. Cell viability was assessed after 24 hours of incubation using the MTT assay, with fractions concentrations ranging from 3.125 to 100 μg/mL. Results are expressed as mean ± SD from three independent experiments.
(TIF)

S4 Table Cell viability (%) of *Khaya senegalensis* subfractions in HaCaT and HDF cells. Cell viability was assessed after 24 hours of incubation using the MTT assay, with subfractions concentrations ranging from 3.125 to 100 μg/mL. Results are expressed as mean ± SD from three independent experiments.
(TIF)

S5 Table Cell viability (%) of *Khaya senegalensis* isolated compounds in HaCaT and HDF cells. Cell viability was assessed after 24 hours of incubation using the MTT assay, with isolated compounds concentrations ranging from 3.125 to 100 μg/mL. Results are expressed as mean ± SD from three independent experiments.
(TIF)

S6 Table Wound closure (%) of *Khaya senegalensis* extracts in HaCaT and HDF cells. Scratch assay results showing wound closure (%) following treatment with extracts at concentrations of 12.5, 25, and 50 µg/mL. Wound closure in HaCaT cells was recorded at 13, 24, 36, and 48 hours, and in HDF cells at 13, 15, 18, 21, and 24 hours. Results are expressed as mean ± SD from three independent experiments. Data were analyzed using one-way ANOVA followed by Tukey’s post hoc test. ^a^*p* < 0.001, ^b^*p* < 0.01, ^c^*p* < 0.05 compared with negative control. Hex = *n-*hexane extract; EA = ethyl acetate extract; MeOH = methanol extract.
(TIF)

S7 Table Wound closure (%) of *Khaya senegalensis* fractions in HaCaT and HDF cells. Scratch assay results showing wound closure (%) following treatment with fractions at concentrations of 12.5, 25, and 50 µg/mL. Wound closure in HaCaT cells was recorded at 13, 24, 36, and 48 hours, and in HDF cells at 13, 15, 18, 21, and 24 hours. Results are expressed as mean ± SD from three independent experiments. Data were analyzed using one-way ANOVA followed by Tukey’s post hoc test. ^a^*p* < 0.001, ^b^*p* < 0.01, ^c^*p* < 0.05 compared with negative control.
(TIF)

S8 Table Wound closure (%) of *Khaya senegalensis* subfractions in HaCaT and HDF cells. Scratch assay results showing wound closure (%) following treatment with subfractions at concentrations of 12.5, 25, and 50 µg/mL. Wound closure in HaCaT cells was recorded at 13, 24, 36, and 48 hours, and in HDF cells at 13, 15, 18, 21, and 24 hours. Results are expressed as mean ± SD from three independent experiments. Data were analyzed using one-way ANOVA followed by Tukey’s post hoc test. ^a^*p* < 0.001, ^b^*p* < 0.01, ^c^*p* < 0.05 compared with negative control.
(TIF)

S9 Table Wound closure (%) of isolated compounds in HaCaT and HDF cells. Scratch assay results showing wound closure (%) following treatment with isolated compounds at concentrations of 12.5, 25, and 50 µg/mL. Wound closure in HaCaT cells was recorded at 13, 24, 36, and 48 hours, and in HDF cells at 13, 15, 18, 21, and 24 hours. Results are expressed as mean ± SD from three independent experiments. Data were analyzed using one-way ANOVA followed by Tukey’s post hoc test. ^a^*p* < 0.001, ^b^*p* < 0.01, ^c^*p* < 0.05 compared with negative control.
(TIF)

S6 Fig Microscopy images of wound closure in HaCaT cells treated with *Khaya senegalensis* isolated compounds. Images of the wounded area treated with different concentrations of samples (12.5, 25 and 50 µg/mL) were captured using an inverted microscope at 4 × magnification at 0, 12, 24, 36, and 48 hours.
(TIF)

S7 Fig Microscopy images of wound closure in HaCaT cells treated with positive and negative control. Images of the wounded area treated with different concentrations of samples (12.5, 25 and 50 µg/mL) were captured using an inverted microscope at 4 × magnification at 0, 12, 24, 36, and 48 hours.
(TIF)

S8 Fig Microscopy images of wound closure in HDF cells treated with *Khaya senegalensis* isolated compounds. Images of the wounded area treated with different concentrations of samples (12.5, 25 and 50 µg/mL) were captured using an inverted microscope at 4 × magnification at 0, 12, 15, 18, 21, and 48 hours.
(TIF)

S9 Fig Microscopy images of wound closure in HDF cells treated with positive and negative control in HDF cells. Images of the wounded area treated with different concentrations of samples (12.5, 25 and 50 µg/mL) were captured using an inverted microscope at 4 × magnification at 0, 12, 15, 18, 21, and 48 hours.
(TIF)

S10 Table Two-dimensional and three-dimensional diagrams of protein-ligand interactions. Binding poses and key interactions between the isolated compounds (β-sitosterol, stigmasterol) and the wound healing targets TNF-α, GSK-3β, and TGF-β1. The analysis reveals interactions including conventional hydrogen bonds, pi-sigma, pi-alkyl, alkyl, and van der Waals forces, which stabilize the ligand-protein complexes.
(PDF)
