# Supplementary figures and images for: Bioassay-guided fractionation and identification of wound healing active compounds from Khaya senegalensis leaves
Source: PLoS One. 2026 Feb 2;21(2):e0339051. doi: 10.1371/journal.pone.0339051 (PMC12863557; doi:10.1371/journal.pone.0339051)

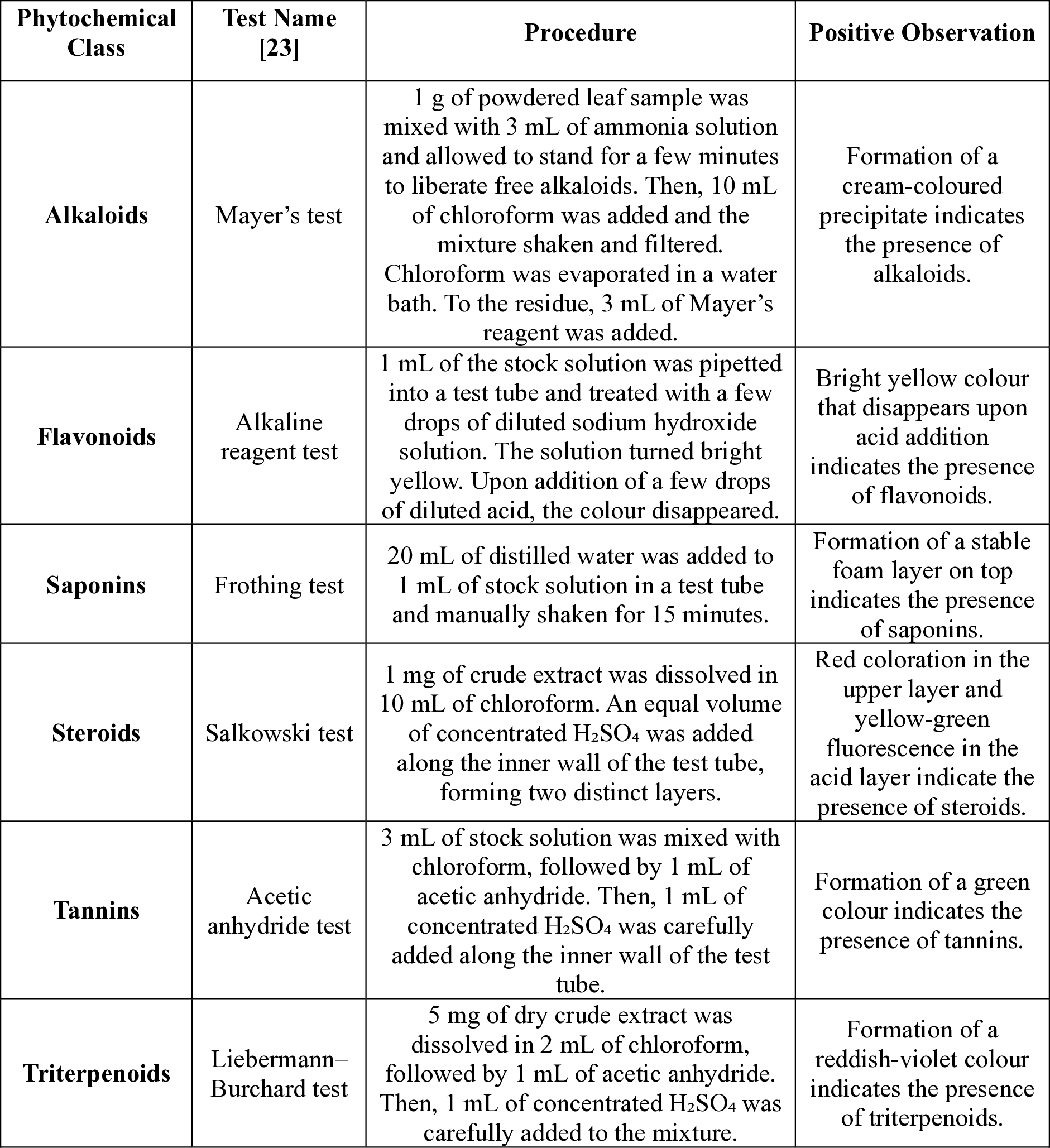

Supplement: S1 Table — (TIF) [file pone.0339051.s001.TIF]

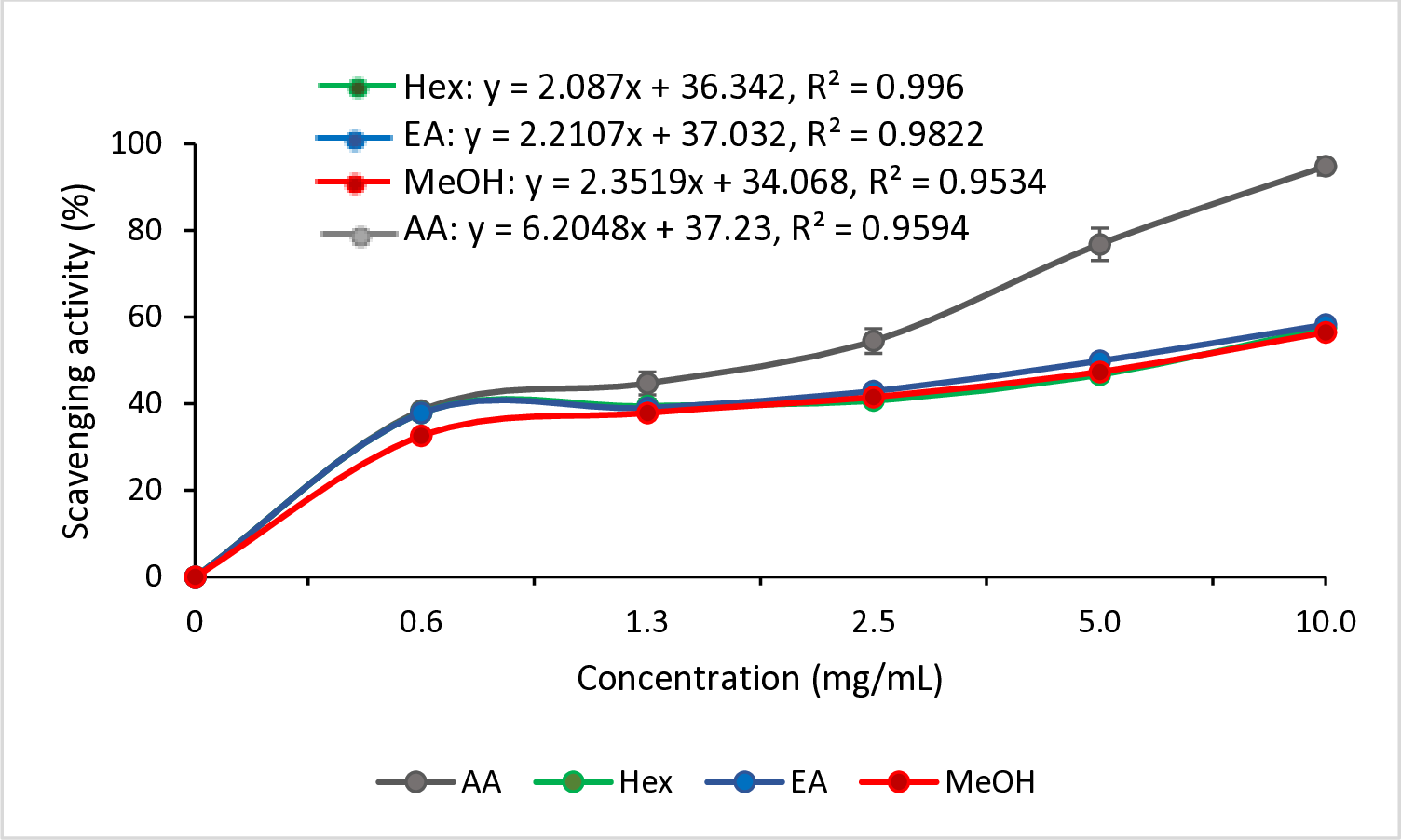

Supplement: S1 Fig — The scavenging activity (%) was plotted against the sample concentration (mg/mL). The IC₅₀ values for each sample were determined from the linear regression equations of the respective curves. Results are expressed as mean ± SD from three independent experiments; error bars indicate SD. Hex = n-hexane extract; EA = ethyl acetate extract; MeOH = methanol extract, AA = ascorbic acid. (TIF) [file pone.0339051.s002.TIF]

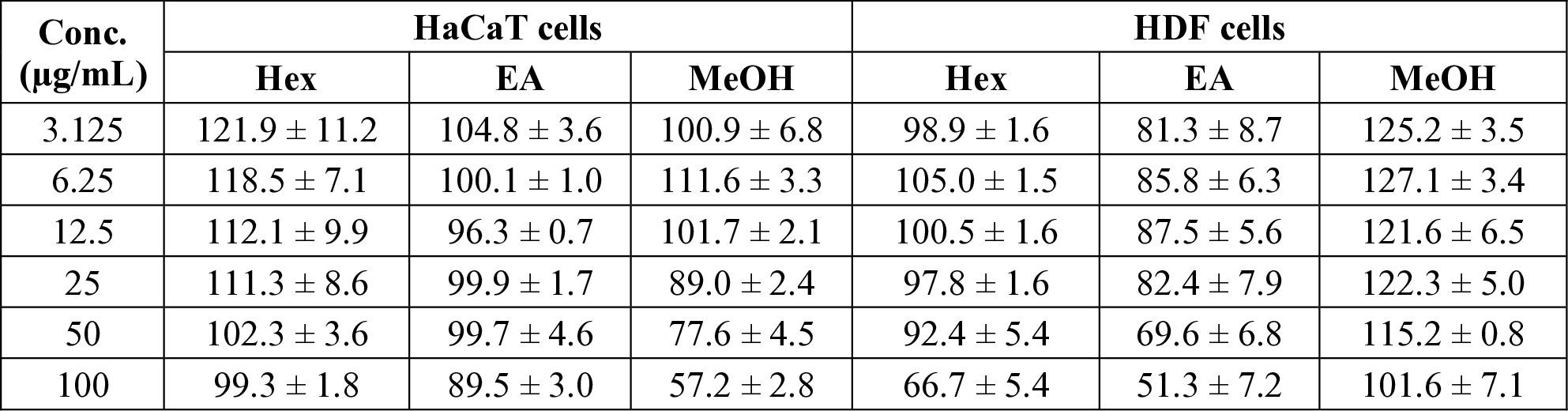

Supplement: S2 Table — Cell viability was assessed after 24 hours of incubation via the MTT assay, with extract concentrations ranging from 3.125 to 100 μg/mL. Results are expressed as mean ± SD from three independent experiments. Hex = n-hexane extract; EA = ethyl acetate extract; MeOH = methanol extract. (TIF) [file pone.0339051.s007.TIF]

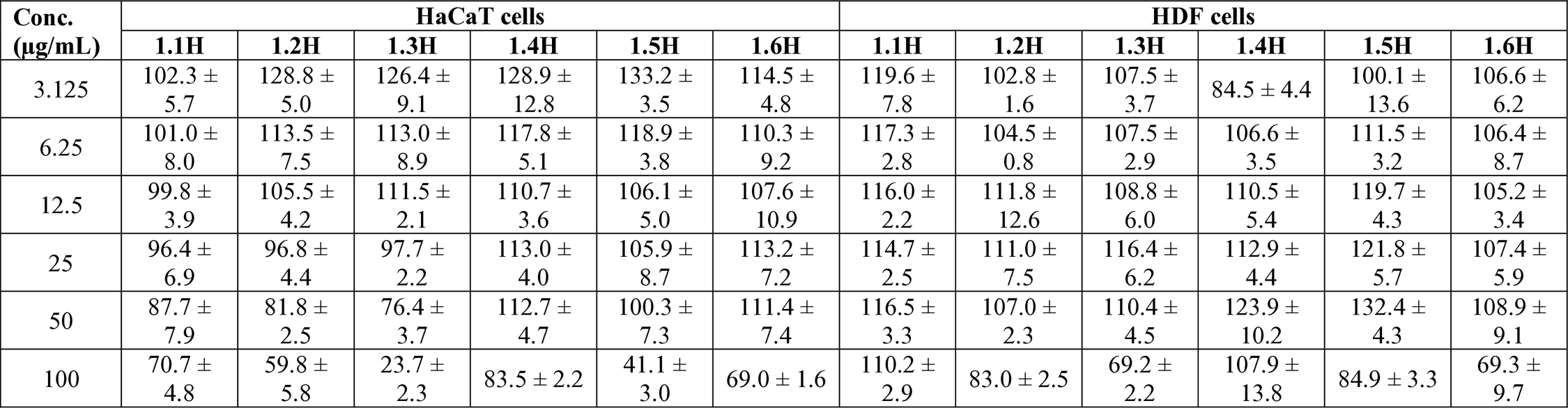

Supplement: S3 Table — Cell viability was assessed after 24 hours of incubation using the MTT assay, with fractions concentrations ranging from 3.125 to 100 μg/mL. Results are expressed as mean ± SD from three independent experiments. (TIF) [file pone.0339051.s008.TIF]

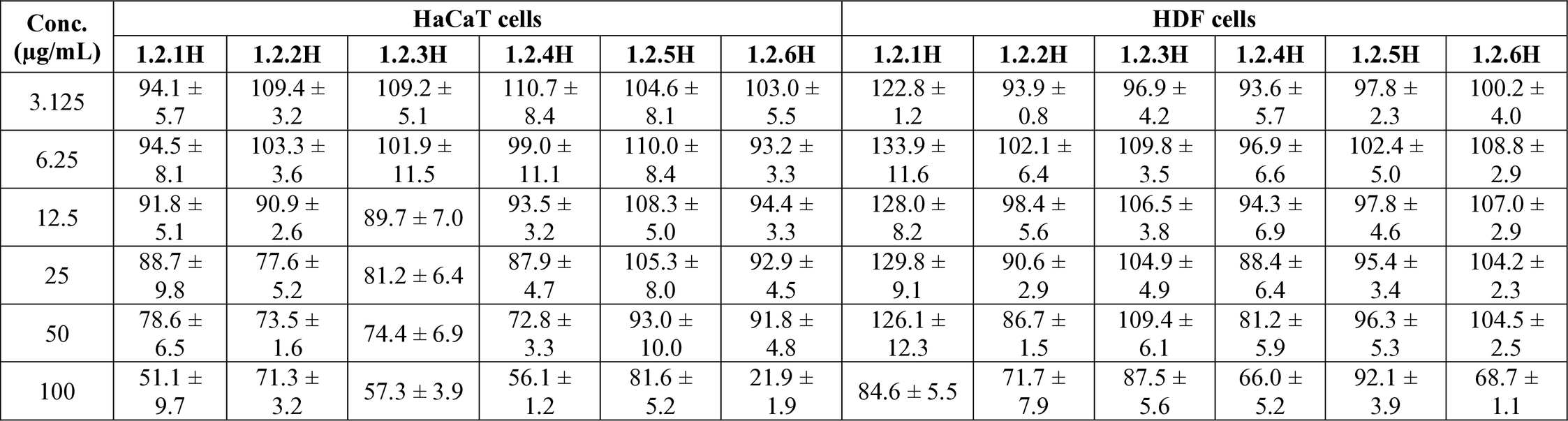

Supplement: S4 Table — Cell viability was assessed after 24 hours of incubation using the MTT assay, with subfractions concentrations ranging from 3.125 to 100 μg/mL. Results are expressed as mean ± SD from three independent experiments. (TIF) [file pone.0339051.s009.TIF]

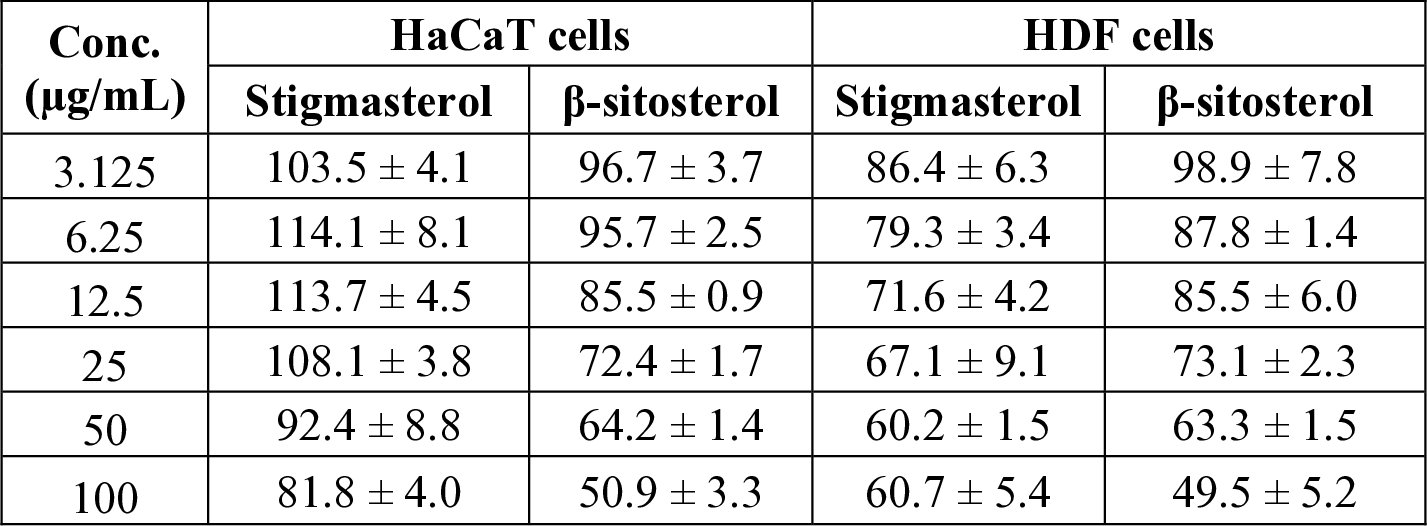

Supplement: S5 Table — Cell viability was assessed after 24 hours of incubation using the MTT assay, with isolated compounds concentrations ranging from 3.125 to 100 μg/mL. Results are expressed as mean ± SD from three independent experiments. (TIF) [file pone.0339051.s010.TIF]

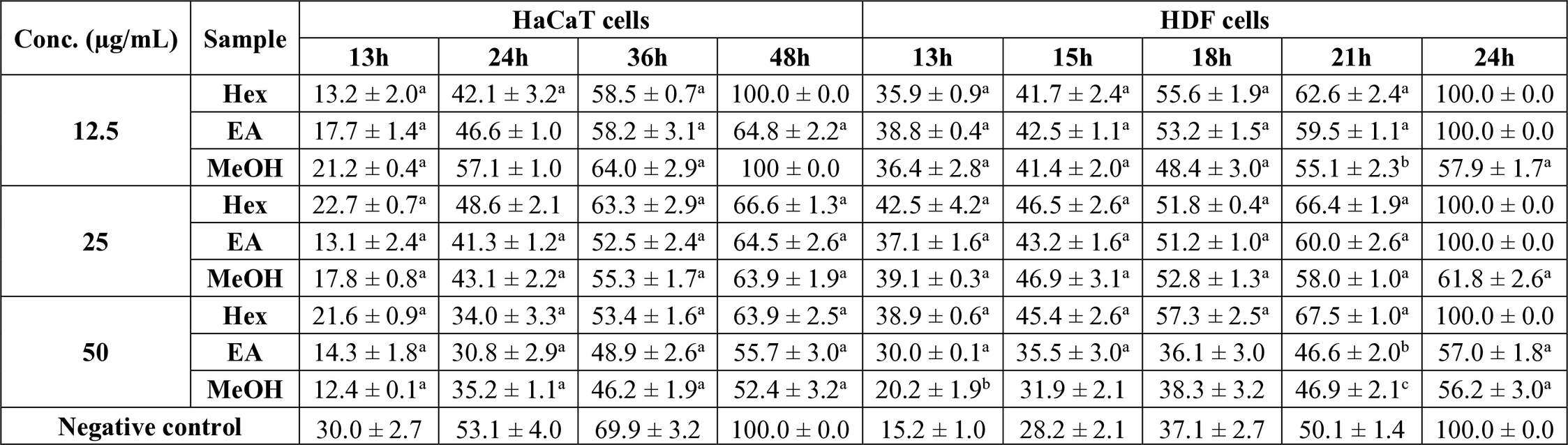

Supplement: S6 Table — Scratch assay results showing wound closure (%) following treatment with extracts at concentrations of 12.5, 25, and 50 µg/mL. Wound closure in HaCaT cells was recorded at 13, 24, 36, and 48 hours, and in HDF cells at 13, 15, 18, 21, and 24 hours. Results are expressed as mean ± SD from three independent experiments. Data were analyzed using one-way ANOVA followed by Tukey’s post hoc test. ap < 0.001, bp < 0.01, cp < 0.05 compared with negative control. Hex = n-hexane extract; EA = ethyl acetate extract; MeOH = methanol extract. (TIF) [file pone.0339051.s011.TIF]

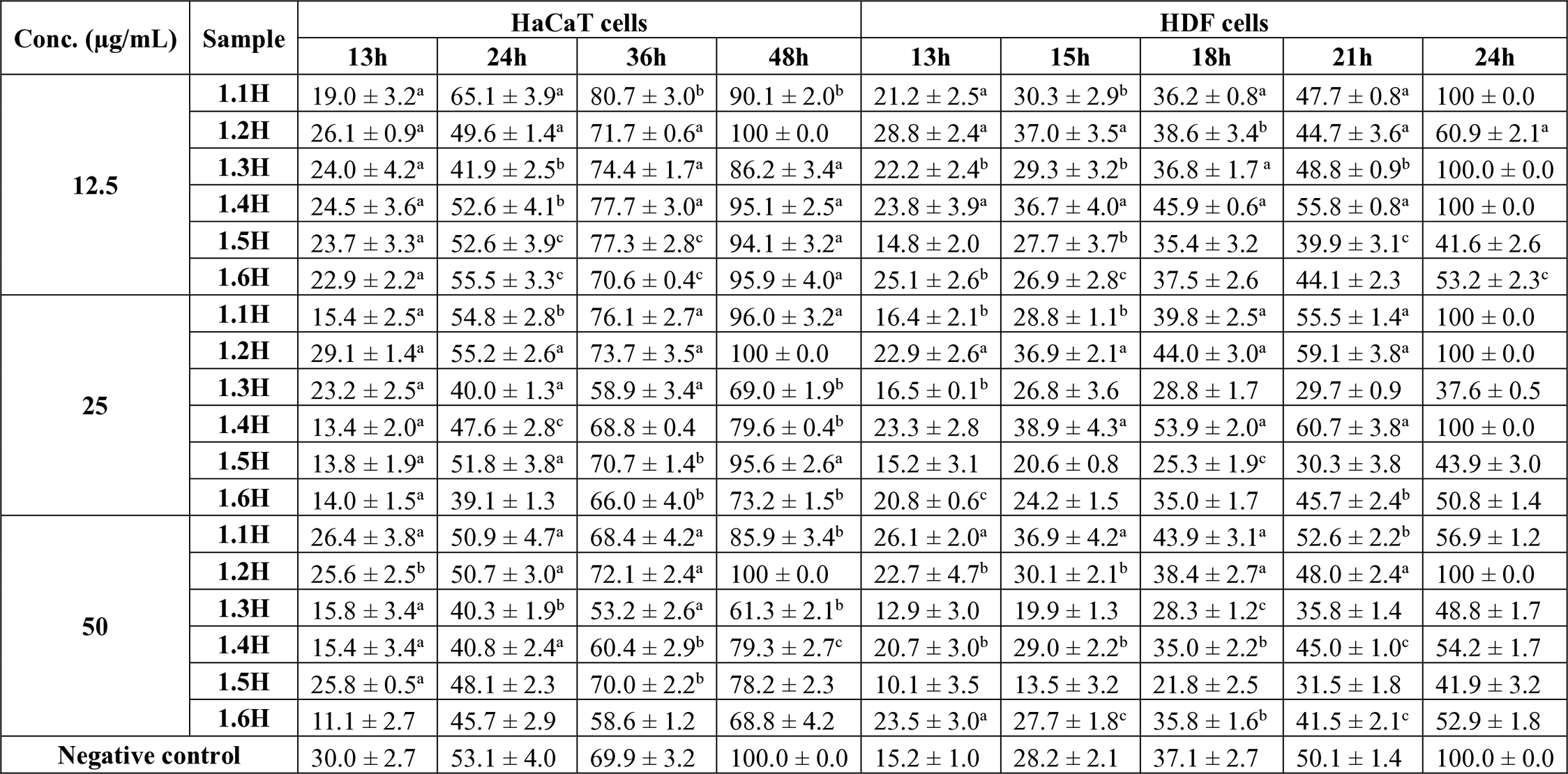

Supplement: S7 Table — Scratch assay results showing wound closure (%) following treatment with fractions at concentrations of 12.5, 25, and 50 µg/mL. Wound closure in HaCaT cells was recorded at 13, 24, 36, and 48 hours, and in HDF cells at 13, 15, 18, 21, and 24 hours. Results are expressed as mean ± SD from three independent experiments. Data were analyzed using one-way ANOVA followed by Tukey’s post hoc test. ap < 0.001, bp < 0.01, cp < 0.05 compared with negative control. (TIF) [file pone.0339051.s012.TIF]

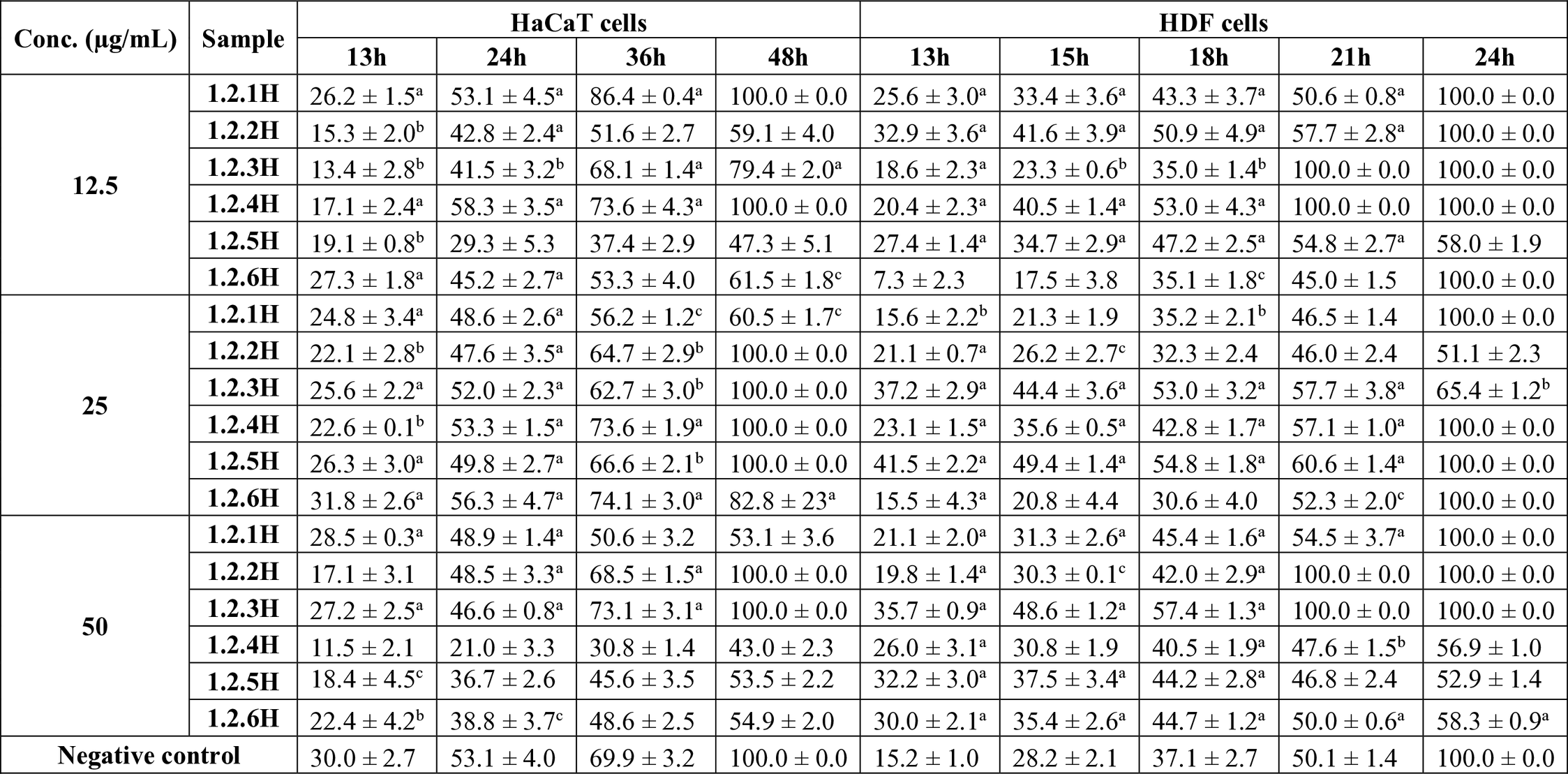

Supplement: S8 Table — Scratch assay results showing wound closure (%) following treatment with subfractions at concentrations of 12.5, 25, and 50 µg/mL. Wound closure in HaCaT cells was recorded at 13, 24, 36, and 48 hours, and in HDF cells at 13, 15, 18, 21, and 24 hours. Results are expressed as mean ± SD from three independent experiments. Data were analyzed using one-way ANOVA followed by Tukey’s post hoc test. ap < 0.001, bp < 0.01, cp < 0.05 compared with negative control. (TIF) [file pone.0339051.s013.TIF]

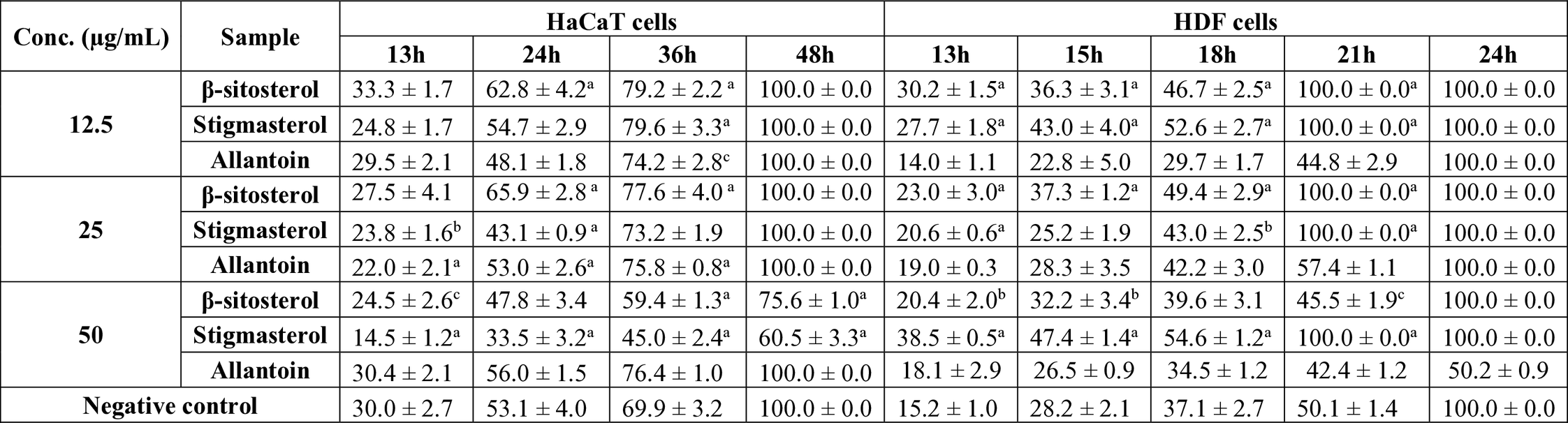

Supplement: S9 Table — Scratch assay results showing wound closure (%) following treatment with isolated compounds at concentrations of 12.5, 25, and 50 µg/mL. Wound closure in HaCaT cells was recorded at 13, 24, 36, and 48 hours, and in HDF cells at 13, 15, 18, 21, and 24 hours. Results are expressed as mean ± SD from three independent experiments. Data were analyzed using one-way ANOVA followed by Tukey’s post hoc test. ap < 0.001, bp < 0.01, cp < 0.05 compared with negative control. (TIF) [file pone.0339051.s014.TIF]

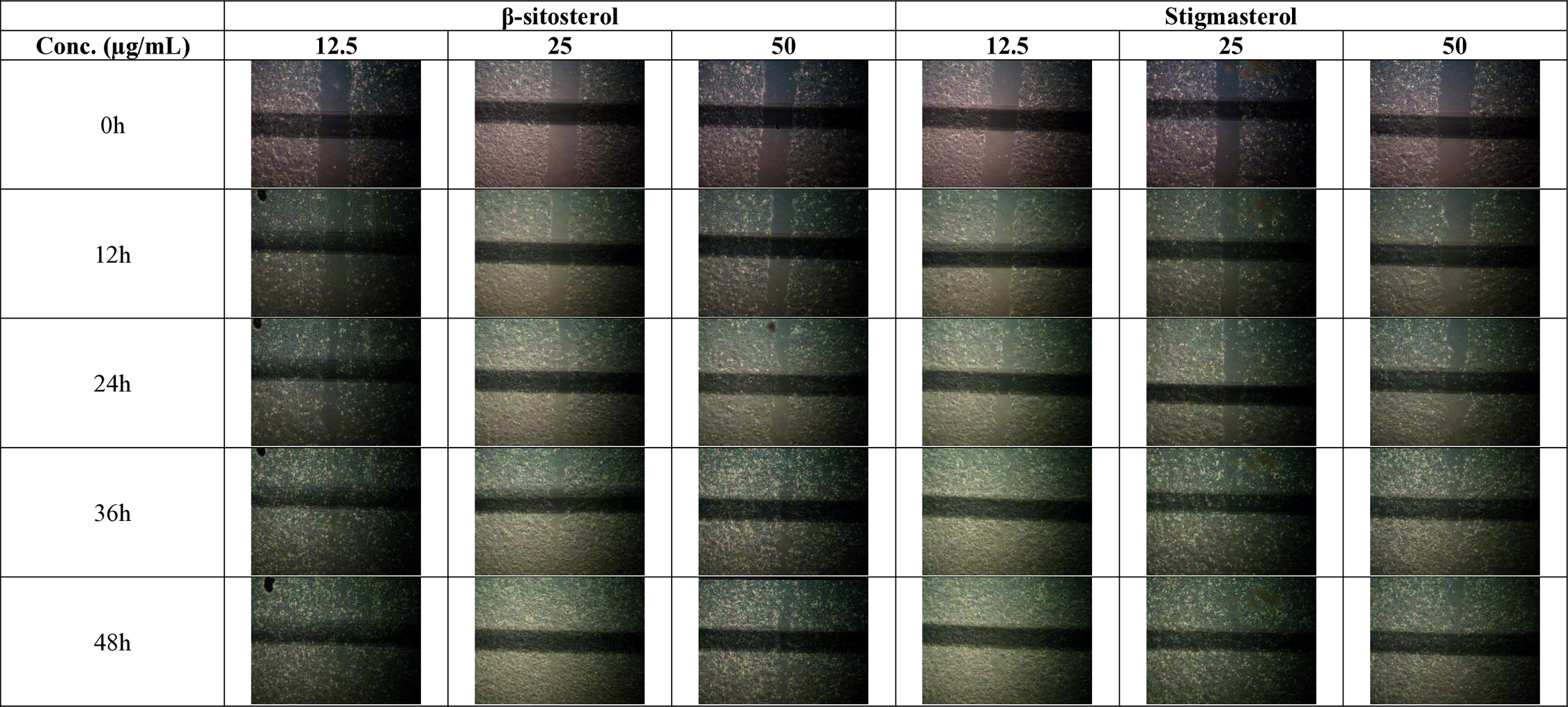

Supplement: S6 Fig — Images of the wounded area treated with different concentrations of samples (12.5, 25 and 50 µg/mL) were captured using an inverted microscope at 4 × magnification at 0, 12, 24, 36, and 48 hours. (TIF) [file pone.0339051.s015.TIF]

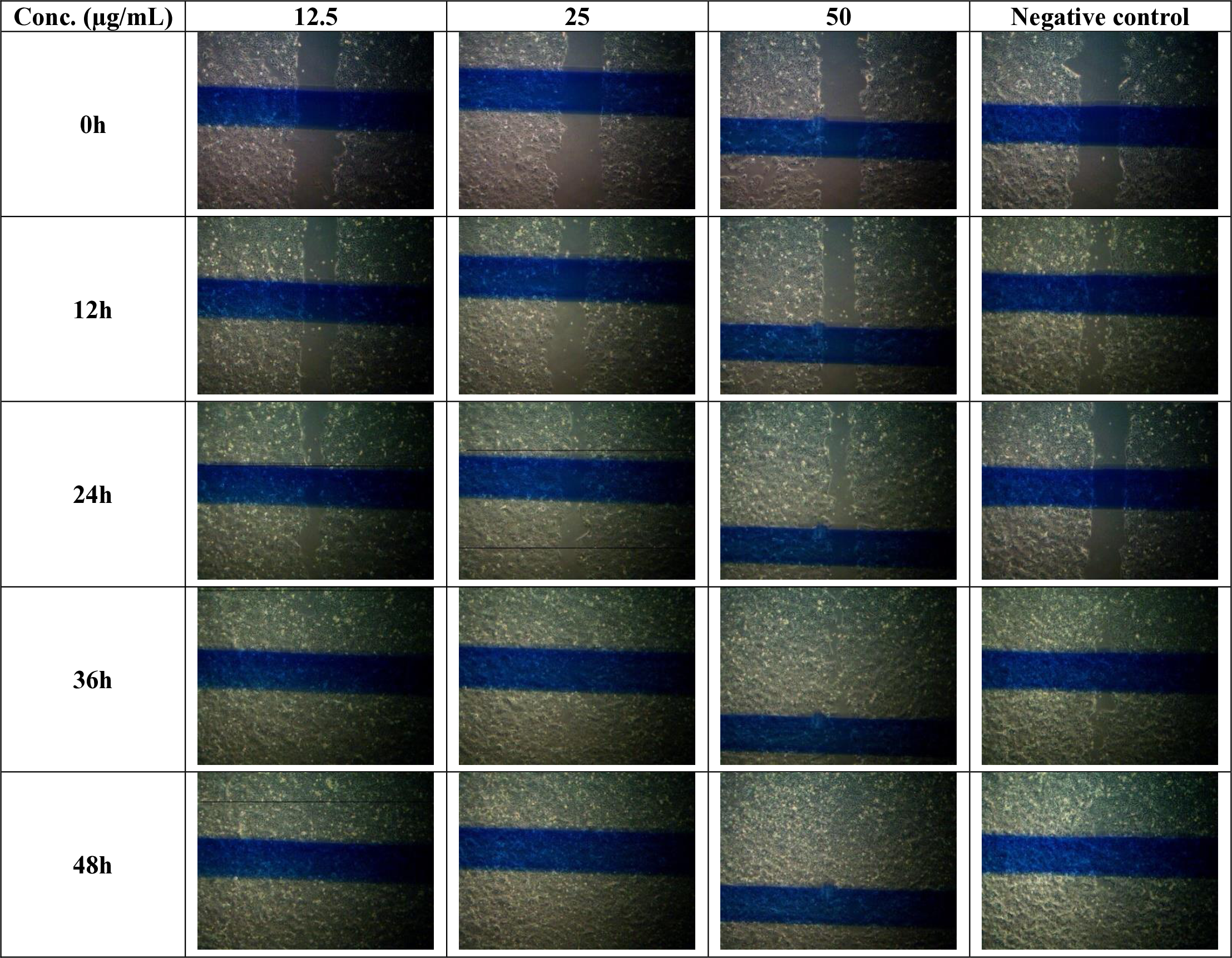

Supplement: S7 Fig — Images of the wounded area treated with different concentrations of samples (12.5, 25 and 50 µg/mL) were captured using an inverted microscope at 4 × magnification at 0, 12, 24, 36, and 48 hours. (TIF) [file pone.0339051.s016.TIF]

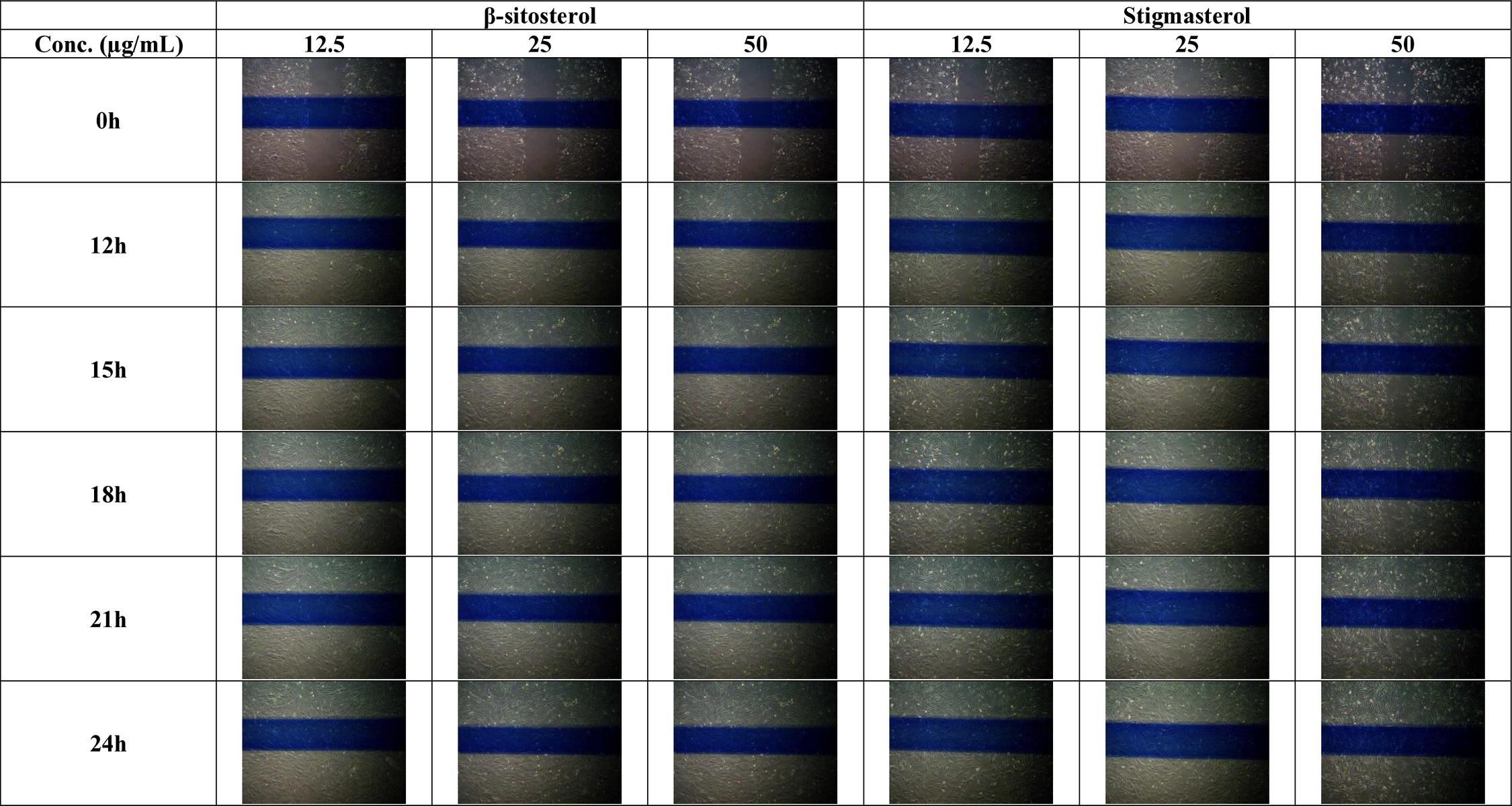

Supplement: S8 Fig — Images of the wounded area treated with different concentrations of samples (12.5, 25 and 50 µg/mL) were captured using an inverted microscope at 4 × magnification at 0, 12, 15, 18, 21, and 48 hours. (TIF) [file pone.0339051.s017.TIF]

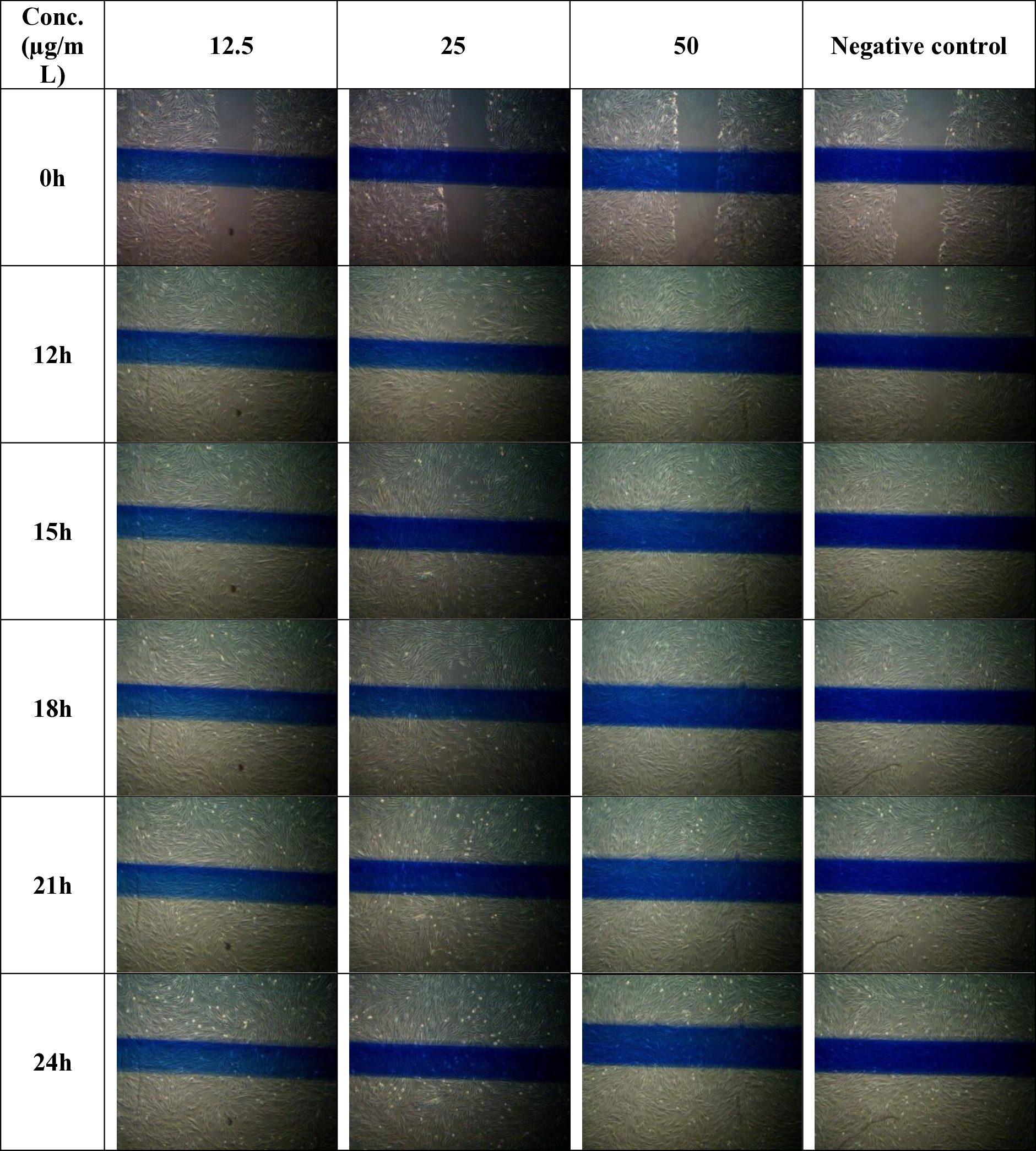

Supplement: S9 Fig — Images of the wounded area treated with different concentrations of samples (12.5, 25 and 50 µg/mL) were captured using an inverted microscope at 4 × magnification at 0, 12, 15, 18, 21, and 48 hours. (TIF) [file pone.0339051.s018.TIF]
