# Supplementary material for: Bioassay-guided fractionation and identification of wound healing active compounds from Khaya senegalensis leaves
Source: PLoS One. 2026 Feb 2;21(2):e0339051. doi: 10.1371/journal.pone.0339051 (PMC12863557; doi:10.1371/journal.pone.0339051)
Supplement: S10 Table — Binding poses and key interactions between the isolated compounds (β-sitosterol, stigmasterol) and the wound healing targets TNF-α, GSK-3β, and TGF-β1. The analysis reveals interactions including conventional hydrogen bonds, pi-sigma, pi-alkyl, alkyl, and van der Waals forces, which stabilize the ligand-protein complexes. (PDF) [file pone.0339051.s019.pdf]

<table>
<tr><th>Protein</th><th>Sample</th><th>2-D</th><th>3-D</th></tr>
<tr><td rowspan="2">2AZ5</td><td>β-sitosterol</td><td>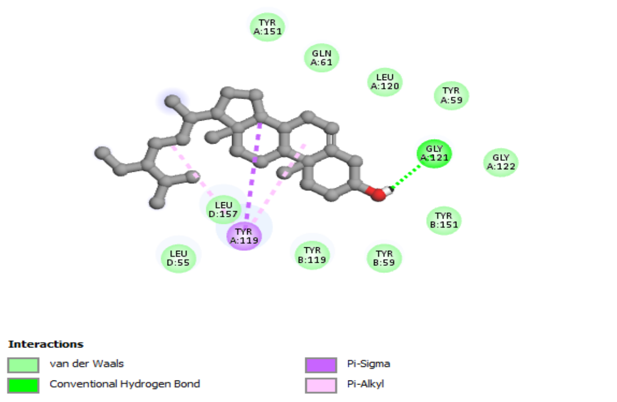
</td><td>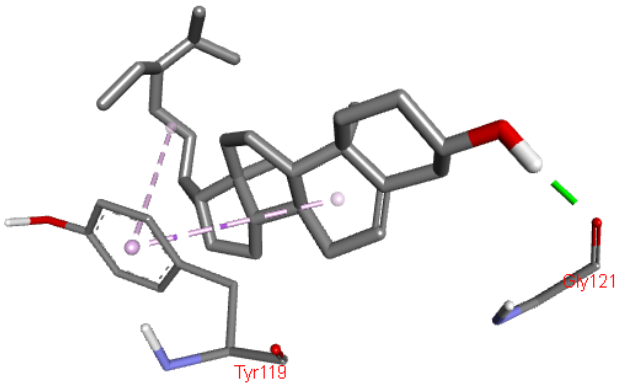
</td></tr>
<tr><td>Stigmasterol</td><td>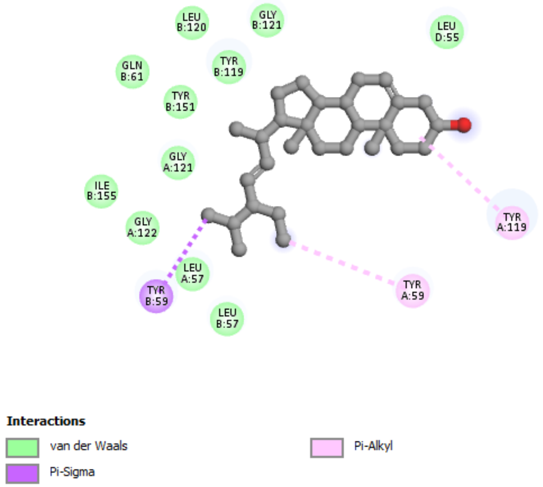
</td><td>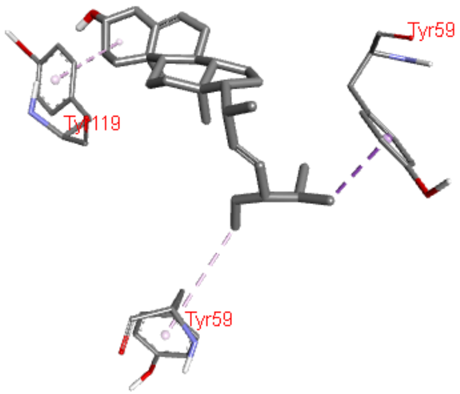
</td></tr>
</table>

<table>
<tr><th>Protein</th><th>Sample</th><th>2-D</th><th>3-D</th></tr>
<tr><td rowspan="2">5K5N</td><td>β-sitosterol</td><td>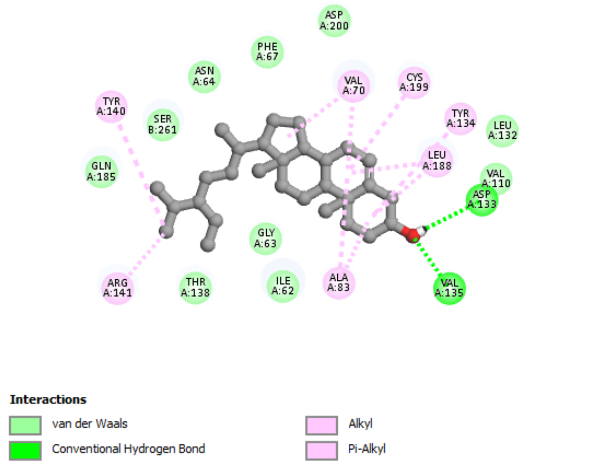
</td><td>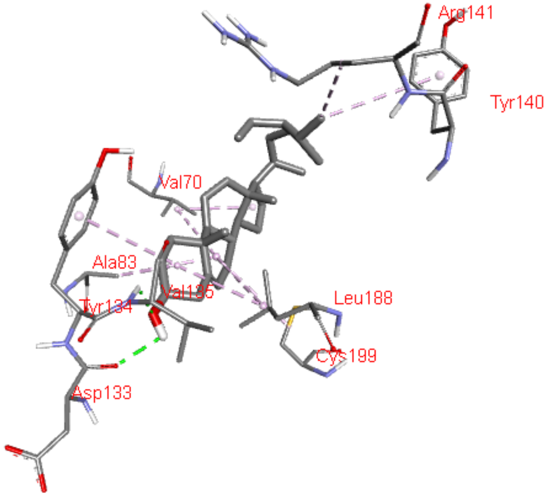
</td></tr>
<tr><td>Stigmasterol</td><td>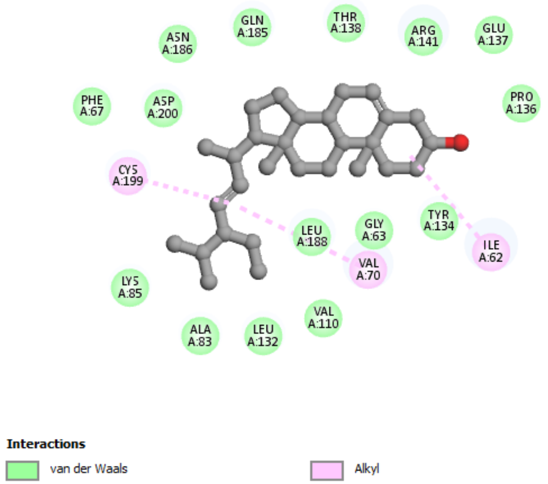
</td><td>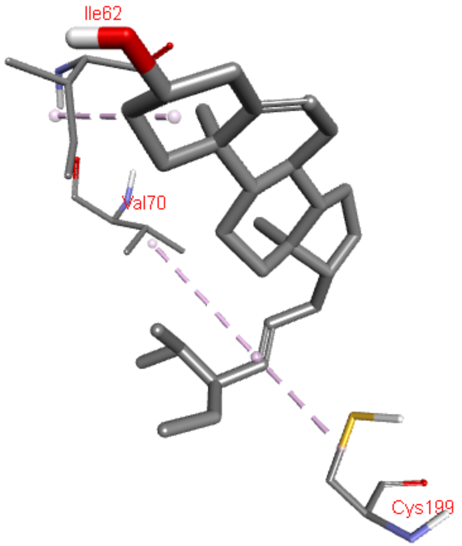
</td></tr>
</table>

<table>
<tr><th>Protein</th><th>Sample</th><th>2-D</th><th>3-D</th></tr>
<tr><td rowspan="2">6B8Y</td><td>β-sitosterol</td><td>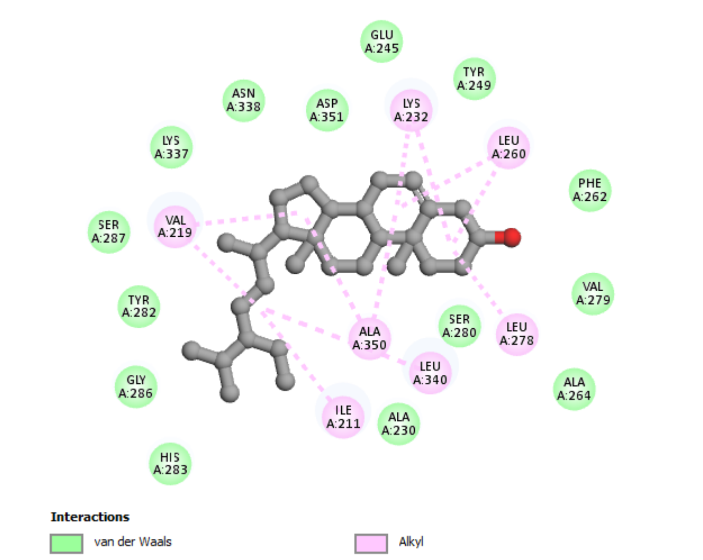
</td><td>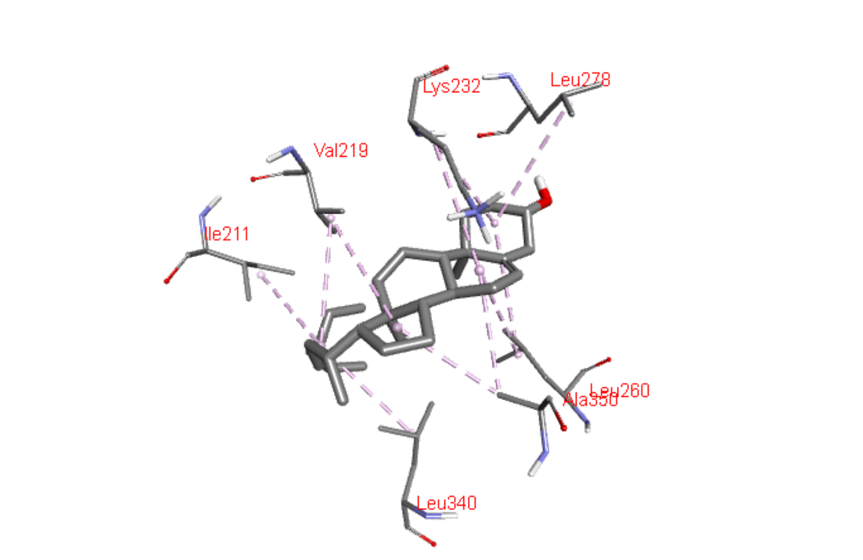
</td></tr>
<tr><td>Stigmasterol</td><td>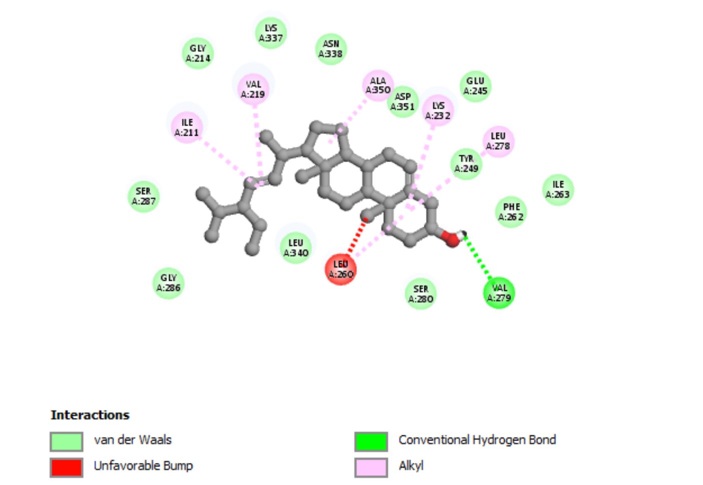
</td><td>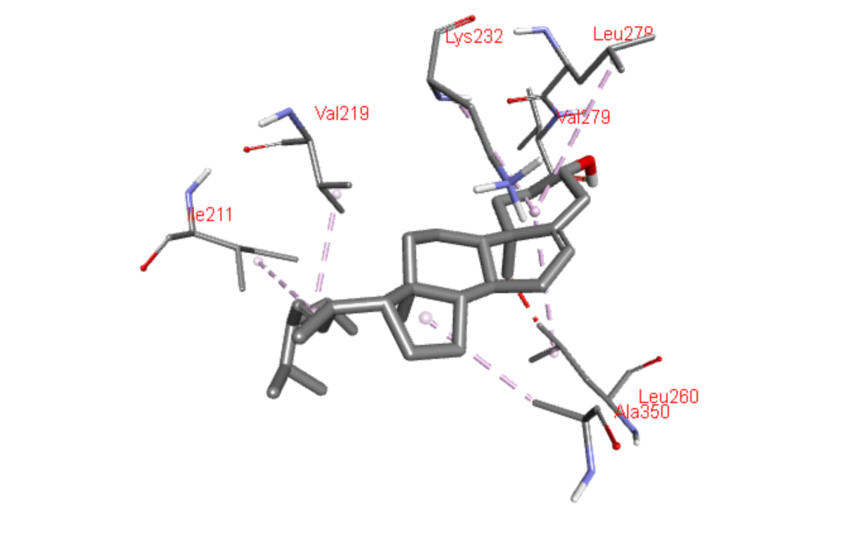
</td></tr>
</table>
